# Eliciting the rubber hand illusion by the activation of nociceptive C and Aδ fibers

**DOI:** 10.1097/j.pain.0000000000003245

**Published:** 2024-05-24

**Authors:** Sara Coppi, Karin B. Jensen, H. Henrik Ehrsson

**Affiliations:** a Departments of Neuroscience and; b Clinical Neuroscience, Karolinska Institutet, Stockholm, Sweden

**Keywords:** Body ownership, Nociception, Nd:YAP laser, Pain, Body representation, Bodily illusion

## Abstract

The coherent perceptual experience of one's own body depends on the processing and integration of signals from multiple sensory modalities, including vision, touch, and proprioception. Although nociception provides critical information about damage to the tissues of one's body, little is known about how nociception contributes to own-body perception. A classic experimental approach to investigate the perceptual and neural mechanisms involved in the multisensory experience of one's own body is the rubber hand illusion (RHI). During the RHI, people experience a rubber hand as part of their own body (sense of body ownership) caused by synchronized stroking of the rubber hand in the participant's view and the hidden participant's real hand. We examined whether the RHI can be elicited by visual and “pure” nociceptive stimulation, ie, without tactile costimulation, and if so, whether it follows the basic perceptual rules of the illusion. In 6 separate experiments involving a total of 180 healthy participants, we used a Nd:YAP laser stimulator to specifically target C and Aδ fibers in the skin and compared the illusion condition (congruent visuonociceptive stimulation) to control conditions of incongruent visuonociceptive, incongruent visuoproprioceptive, and no nociceptive stimulation. The illusion was quantified through direct (questionnaire) and indirect (proprioceptive drift) behavioral measures. We found that a *nociceptive rubber hand illusion* (N-RHI) could be elicited and that depended on the spatiotemporal congruence of visuonociceptive signals, consistent with basic principles of multisensory integration. Our results suggest that nociceptive information shapes multisensory bodily awareness and contributes to the sense of body ownership.

## Abstract 2

Supplemental Digital Content is Available in the Text.

The feeling of owning our bodies is created by combining information from multiple senses, such as nociceptive pain, vision, and proprioception.

## 1. Introduction

Body ownership refers to the immediate perceptual experience of the body as one's own, a fundamental aspect of bodily awareness.^33,34,40^ A classic way to study body ownership in healthy individuals is to use a bodily illusion known as the rubber hand illusion (RHI).^11,33^ The RHI is elicited by synchronously stroking the real hand of the participant, hidden behind a panel, and a rubber hand placed in full view of the participant. A brief period of repeated visuotactile stimulation typically elicits an illusory sensation of the rubber hand being one's own and that it senses the touches one sees.^11,127^ The illusion depends on the spatial and temporal correspondences of visual, tactile, and proprioceptive information^21,24,25,49,73,113^ as well as sensory uncertainty and prior experience.^22,108^ If the signals are sufficiently well matched to a certain degree of congruence, the illusion is triggered, but if the degree of spatial or temporal congruence is too low, the illusion is not elicited.^21,24,54^ Thus, the RHI arises due to the combination of visual, tactile, and proprioceptive information into a coherent multisensory representation of the rubber hand as one's own.^33,34,63,108^

However, surprisingly little is known about how nociceptive signals contribute to the multisensory experience of one's own body. This may seem odd given that nociception provides critical signals about the state of one's own body similar to other senses^92^ and that pain is a quintessential self-related bodily experience.^31,130^ One previous study showed that synchronous stimulation seen and felt concomitant mechanical noxious stimuli (delivered through a sharp pin) could elicit the RHI, but asynchronous stimulation could not^19^; another study showed that nonpainful thermal stimulation modulated the RHI^60^; and a third study reported that the RHI could be elicited through synchronous tactile–thermal–nociceptive stimulation (through a thermod touching the real hand).^23^ However, in these studies, the stimulation of tactile receptors was always concurrent with nociceptive stimulation, so it is unclear whether the reported illusion-related effects were driven mainly by somatosensory processes through cutaneous mechanoreceptors (eg, Aβ fibers) or nociceptive signals (C fibers and Aδ fibers).

To address this question, we used a contactless radiant heat laser to selectively stimulate the skin receptors that primarily convey nociceptive and thermal inputs—C fibers and Aδ fibers—*without* activating tactile mechanoreceptors (ie, Aβ fibers)^9,12,28,52,53,99^ and examined whether this type of “pure” nociceptive stimulation can be used to trigger the RHI. In 6 separate experiments with healthy participants (n = 30 each), nociceptive stimuli were delivered to the participant's real hand in synchrony with a visual stimulus (red light from a diode laser) presented on the rubber hand; control conditions used spatially and temporally incongruent visuonociceptive stimulation, incongruent visuoproprioceptive information, and no nociceptive stimulation. The illusion was quantified psychometrically through questionnaire ratings and an indirect behavioral measure that registered the shift in perceived hand location toward the rubber hand (proprioceptive drift).^11,127^ We hypothesized that a nociceptive RHI (N-RHI) would be elicited and that it would depend on congruent visuonociceptive and visuoproprioceptive information, consistent with the multisensory principles of body ownership.^11,21,25,30,33,34,54,63,126^

## 2. Materials

### 2.1. Rationale, hypothesis, and experimental design

All experiments tested the overall hypothesis that congruent visual and nociceptive signals would lead to elicitation of the RHI and that the N-RHI should adhere to similar temporal and spatial multisensory integration rules as the classic RHI. The classic visuotactile rubber hand illusion is typically examined by comparing an illusion condition with congruent visual and somatosensory stimulation (eg, seen and felt brushstrokes) to a control with incongruent stimulation in otherwise equivalent conditions.^10,11,34,35,127^ Thus, the key comparison in this study was between congruent and incongruent visual and nociceptive stimulation conditions with repeated brief laser light stimuli presented on the rubber hand and brief nociceptive laser stimulation on the real hand (see further below). To quantify the N-RHI we used, the 2 most commonly used measures in the RHI literature, questionnaire ratings of the subjective illusion experience and changes in perceived hand position sense toward the location of the rubber hand using an objective behavioral measure (proprioceptive drift).^11,34,123^ The study used 3 experimental designs (1, 2, and 3), and we registered questionnaire ratings (A) and proprioceptive drift (B) in separate experiments; thus, we collected data in 6 separate experiments conducted with different groups of naive participants (labeled Experiments 1A to Experiments 3B). The experiments were conducted in chronological order of which they are reported in the text.

Specifically, in Experiments 1A and 1B, we contrasted congruent vs incongruent visuonociceptive stimulation. We predicted a stronger subjective RHI in Experiment 1A and a greater proprioceptive drift in Experiment 1B for the congruent condition. Experiments 2A and 2B compared the congruent illusion condition with a control condition, commonly used in the RHI literature^35,54,73,127^: here, the rubber hand was presented in an anatomically incongruent position, eliminating the illusion, while maintaining identical congruent visual and nociceptive laser stimulation. We expected stronger subjective illusion ratings and proprioceptive drift in the congruent condition. Finally, Experiments 3A and 3B replicated the congruent vs incongruent visuonociceptive comparisons from Experiments 1A and 1B and introduced 2 additional control conditions. In these controls, the real hand received no nociceptive stimulation, with participants either observing the rubber hand with visual stimulation or simply viewing the unstimulated rubber hand. We expected the most pronounced N-RHI in the congruent condition.

### 2.2. Participants

The sample size of 30 participants per experiment was determined before the study commenced based on the previous literature about the RHI.^19^ We did not perform an a priori power analysis. Because we investigate a novel effect, no relevant previous studies could be used in a meaningful power analysis, we reasoned. Instead, we chose a predetermined sample size of 30 to ensure a semicounterbalance of the order of our experimental conditions. Notably, rubber hand illusion studies using questionnaires and proprioceptive drift commonly employ a sample size ranging from 20 to 30 participants. Thus, we reasoned that our chosen sample size would be appropriate. Participants who did not fulfil the inclusion criteria or did not complete the entire experiment due to either sensitization or technical failure were replaced with a new participant until we reached the target sample. The inclusion criteria were as follows: participants were fully healthy (Experiments 1A-3B), aged between 18 and 50 years (Experiments 1A-3B), never had skin diseases (Experiments 1A-3B), had no scars or tattoos on the planned stimulation area (Experiments 1A-3B), and had not participated in a bodily illusion experiment before (Experiments 1A-3B). Additionally, participants had not taken any painkillers, medical or recreational drugs, or alcohol within 24 hours prior to the experiment (Experiments 2A-3B), had never experienced abnormal skin reactions to brief sunlight exposure (Experiments 2A-3B), and had not been exposed to sunlight or a solarium during the 3 weeks prior to the experiment (Experiments 2A-3B). Thus, different groups of 30 naÏve volunteers took part in each of the 6 experiments (Table 1). To assess handedness, we used the Edinburgh Inventory^95^ before the beginning of the experiment. Participants were recruited through online advertisements and physical posters at university campuses.

**Table 1:** Descriptive data.

Experiment	Conditions	Measurement	Block (N)	Participants	Fluence mJ/mm^2^	VAS during pain calibration
1A	Congruent vs incongruent	Questionnaire	2	30 = 16 M—14 F M_age_ = 26.8 SD_age_ = ±3.49 24R ∼ 6L	M = 68.79 SD = ±16.06 Min = 32.497 Max = 97.491	M = 14.13 SD = ±8.67 Min = 2.9 Max = 34.7
1B		Proprioceptive drift	6	30 = 11 M—19 F M_age_ = 24.7 SD_age_ = ±2.75 30 R	M = 64.778 SD = ±18.58 Min = 25.998 Max = 97.491	M = 11.18 SD = ±7.64 Min = 2.4 Max = 35.2
2A	Congruent vs rotated	Questionnaire	2	30 = 16 M—14 F M_age_ = 24.26 SD_age_ = ±2.86 25R ∼ 3L ∼ 2A	M = 60.228 SD = ±20.9 Min = 25.998 Max = 110.49	M = 14.71 SD = ±4.28 Min = 7.8 Max = 24.5
2B		Proprioceptive drift	6	30 = 9 M—21 F M_age_ = 26 SD_age_ = ±5.46 27R ∼ 3L	M = 85.792 SD = ±18.883 Min = 58.495 Max = 116.989	M = 18.34 SD = ±9.42 Min = 5 Max = 40.9
3A	Congruent vs incongruent vs hand vs light	Questionnaire	4	30 = 14 M—16 F M_age_ = 27.06 SD_age_ = ±5.72 26R ∼ 3L ∼ 1A	M = 59.578 SD = ±21.527 Min = 25.998 Max = 97.491	M = 16.76 SD = ±6.04 Min = 1.4 Max = 31.1
3B		Proprioceptive drift	12	30 = 14 M—16 F M_age_ = 26.97 SD_age_ = 5.275 29R ∼ 1A	M = 56.762 SD = ±19.61 Min = 25.998 Max = 103.991	M = 15.17 SD = ±2.45 Min = 10.9 Max = 19.3

Descriptive statistics of the fluence energy in each experiment and the amount of pain during the calibration phase for each experiment are shown. The visual analog scale ranged from 0 to 100.

A, ambidextrous; L, left-handed; M, mean; R, right handed; SD, standard deviation.

All experiments were approved by the Swedish Ethics Review Authority. All subjects provided written consent to take part in the study, and they received a small monetary compensation (150-250 SEK before taxation) or movie ticket for their participation.

### 2.3. Nociceptive rubber hand illusion setup

We developed a nociceptive version of the classical RHI paradigm.^11^ The participant was seated in front of a table. On the table, a lifelike cosmetic prosthetic left hand filled with plaster (the rubber hand) (Fillauer Europe AB, PVC color Y02, LT) was placed in the participant's field of view, approximately 21 cm to the left of their body midline, in a position so that it visually resembled their own hand. A black cloth covered the proximal end of the rubber hand and the participant's shoulder so that the participant could not see the gap between the rubber hand and their body. The participant's real left hand was hidden behind an occluding screen and placed on the table between the rubber hand and the real hand at a 90° angle in the sagittal plane, resting in a relaxed position on the table. The distance between the real left index finger and the rubber hand's index finger was 17.5 cm (in all experiments), which is close enough (within peripersonal space) for the potential induction of the RHI.^17,73^ To elicit the illusion, we delivered synchronized brief visual stimuli on the rubber hand (ie, red dots from a diode laser) and nociceptive laser stimuli on the real hand. To ensure that the visual and nociceptive stimuli would be as synchronous, the visual stimuli were delivered with 60 milliseconds of delay as compared with the nociceptive input in line with previous studies that has found that this delay creates the impression of simultaneity.^80,81,131^

### 2.4. Nociceptive laser stimulation

Noxious stimuli were delivered by the use of a Nd:YAP laser (Stimul 1340 Neurolas, Deka, Calenzano, Italy) that operates with a wavelength of 1.34 µm. The laser beam was transmitted through a 10-m optic fiber. This laser has been previously used to study pain^66,78,93^ and thermosensation without Aβ-fiber activation.^119^ Radiant heat lasers, including and Nd:YAP and CO_2_ lasers, have been extensively studied for their ability to activate nociceptive pathways in humans. This is typically assessed through the measurement of laser-evoked potentials (LEPs) using electroencephalography (EEG) and verbal pain reports.^12–16^ Laser-evoked potentials are considered the gold standard for investigating nociceptive pathways.^27,132^ Using this and other methods, it has been shown that the Nd:YAP laser selectively activates C and Aδ fibers in humans.^3,28,52,67,116,124^ C and Aδ fibers have different conduction velocities. Aδ fibers have a faster conduction velocity (15 m/second) and carry the so-called first pain, which refers to a pinpricking pain sensation. C fibers have a slower conduction velocity (0.5-1.5 m/second) and carry the so-called second pain, which refers to a burning pain sensation.^72,77^ The diameter was set at 7 mm (spot area = ∼ 38 mm^2^), and the duration was 7 milliseconds. To avoid touches by the laser handpiece, we kept the handpiece at a distance of approximately 0.5 cm from the hand. This short distance did not significantly change the diameter of the resulting stimulation spot on the hand dorsum. The intensity of the laser stimulation was calibrated (the calibration procedure is described in the Supplemental Digital Content, http://links.lww.com/PAIN/C39) for each participant to be safe and to correspond to mild subjective pain. The average fluence across the 6 experiments was 65.987 (±21.392 SD) mJ/mm^2^ (Table 1). This energy is associated with nociceptive activation, specifically C and Aδ nociceptor activation.^39,51,128^ In addition, pulses of intensity at 46-76 mJ/mm^2^ are associated with an increase in human skin temperature to 48°C, which in turn is associated with reports of a pinprick sensation.^28^ The Nd:YAP laser was controlled through a program developed with the software C++. The experimenter and the participant always wore special protective glasses (000-G0140-RETR-21, PROTECT Laserschutz GmbH) when the laser was turned on to avoid accidental damage to the retina.

### 2.5. Visual stimulation of the rubber hand

The visual stimulus (red light) on the rubber hand was delivered by a low-intensity (nonnociceptive) diode laser (VLM-650-01 PT, Laser Diode 650 nm, 1 MW, 10.4 mm DIA); the light had a similar circular shape and color as the pointer produced by the nociceptive laser. The duration of the visual red-light stimulus of the diode laser was 130 milliseconds, and its diameter on the rubber hand was 7 mm. These parameters were determined in pilot experiments to resemble the nociceptive laser visual impression and to be easy for the participants to see. The laser light was controlled through a program developed with the software C++, the same software used for the Nd:YAP laser.

### 2.6. Electromyography

In Experiments 3A and 3B, we monitored muscle activity in the participants' real left arm to ensure similar levels of muscular activity in the different experimental conditions. We recorded surface electromyography (EMG) from the left bicep and the left extensor carpi radialis longus (named ‘extensor’ in the results section) using surface electrodes (DE-2.1 single differential electrodes, Bagnoli, Delsys) and the Delsys Bagnoli desktop system (Delsys Inc, Natick, MA). The electrodes were placed on the skin over the belly of the muscle after cleaning the area with alcohol wipes. The reference electrode was placed on the skin over the left hip bone. The EMG signal was recorded through Spike2 software (version 7.04) through a CED Micro1401-3 data acquisition unit (Cambridge Electronic Design Limited, Cambridge, United Kingdom). We then analyzed the EMG data in 2 ways. First, we averaged the EMG activity of each block (120 seconds) to obtain an overall estimate of muscular activity throughout each block (block-average EMG). We compared these values across conditions. Second, we calculated the average EMG activity in a time window of 200 milliseconds after each painful nociceptive laser stimulation (laser-evoked EMG) and compared these values between the nociceptive conditions, that is, congruent vs incongruent. This latter EMG measurement was used to probe putative muscular twitches triggered by nociceptive stimulation, as we hypothesized that it might potentially interfere with the RHI. We needed to remove 1 data set in Experiment 3A and 1 data set in Experiment 3B due to a failure of the system to record the EMG signal, leaving us with 29 data sets for the analysis in each experiment.

### 2.7. Outcome measures: questionnaire and proprioceptive drift

The N-RHI was quantified with questionnaire ratings and a proprioceptive drift task, 2 commonly used measures in previous studies as described above.^11,127^ For the pain calibration procedures and for monitoring the degree of experienced pain during the N-RHI experiments, we used a 0-100 mm visual analog scale (VAS) to quantify perceived pain intensity.^102^ We did not have any specific hypothesis regarding the pain ratings after the various conditions, and these results are reported for purely descriptive purposes (ie, we did not expect the RHI to modulate perceived pain intensity,^88^ although some studies have claimed that such modulation may occur after the induction of bodily illusions, either being a hyperalgesic modulation^115^ or analgesic^75,79^). We also did not have any specific hypothesis regarding the electromyography recordings (EMG) other than that we expected the participants to be similarly relaxed with their left arm under the different conditions; however, because pain may trigger automatic motor defense reactions and twitches that might interfere with the illusion, such as the nociceptive flexion reflex,^117^ we thought that this was relevant to monitor.

#### 2.7.1. Rubber hand illusion questionnaire

In the questionnaire assessment, the participants used a 7-point Likert scale (from −3 strongly disagree to +3 strongly agree) to rate how much they agreed (positive scores) or disagreed (negative scores) with 4 statements about the various key perceptual aspects of the illusion, such as feeling ownership of the rubber hand (S1 and S2) and sensing pain sensations originating from the rubber hand (S5 and S6; Table 2). The questionnaire additionally included 4 control statements (S3, S4, S7, and S8), which served as a quantitative “sanity check” for task compliance and susceptibility to suggestibility effects. We descriptively confirmed that participants gave low scores to these control statements; however, the data were not used in the statistical analyses. The questionnaire statements we used were adapted from the original Botvinick and Cohen questionnaire.^11^ In the questionnaire experiments (Experiments 1A, 2A, and 3A), we measured the subjective experience through questionnaire once per condition.

**Table 2:** Questionnaire.

Statement number	Statement	Statement class	Exp
S1. Hand ownership	It felt as if the rubber hand were my hand	*Illusion* (visuonociceptive–proprioceptive integration)	1a 2a 3a
S2. Hand ownership	It felt as if the rubber hand was part of my body	*Illusion* (visuonociceptive–proprioceptive integration)	1a 2a 3a
S3. Control hand	It felt as if I might have more than one left hand or arm	*Control*	1a 2a 3a
S4. Control hand	It seemed as if my real hand was larger than normal	*Control*	1a 2a 3a
S5. Referral of pain	It seemed as if I were feeling the pain in the location where I saw the light on the rubber hand	*Illusion* (visuonociceptive integration)	1a 3a
S5. Referral of pain	It seemed as if I were feeling the pain on the rubber hand in the location where I saw the light (on the rubber hand)	*Illusion* (visuonociceptive integration)	2a
S6. Referral of pain	It felt as if the painful sensation I felt was caused by the laser light on the rubber hand	*Illusion* (visuonociceptive integration)	1a 2a 3a
S7. Control	It seemed as if the pain came from somewhere between my own hand and the rubber hand	*Control*	1a 2a 3a
S8. Control	It felt as if my hand was cold	*Control*	1a 2a 3a

The statements were presented through a Likert scale questionnaire for each experiment.

#### 2.7.2. Proprioceptive drift

In the proprioceptive drift task, participants manually reported the sensed location of their unseen real left index finger immediately before and immediately after each block of the RHI induction or control condition (see paragraph 3.3 below). Participants moved their right index finger along a ruler placed on the table 8.5 cm over the real hand to indicate the position they felt corresponded to the location of their left index finger. For each block, different starting positions for the right index finger were used so that the participant did not just repeat the same movement. The proprioceptive drift was calculated as the difference between the measurement taken before the start of each condition and the measurement taken after each condition. Positive drift scores (ie, positive difference) corresponded to a change in perceived hand position sense toward the rubber hand.^1,126^ For each condition in the proprioceptive drift experiments (“B”), we measured the proprioceptive drift difference 3 times and averaged it to provide 1 average proprioceptive drift score (mean of the differences) per participant.

### 2.8. Pain visual analog scale ratings

To confirm that participants experienced mild pain in all conditions, we measured pain intensity using a VAS. Perceived pain was assessed through a slider on a 0- to 100-mm VAS after each block (in all experiments). This assessment was performed to control for pain intensity in our analyses of the RHI and to ascertain that pain was stable across the blocks (ie, no sensitization or desensitization). The VAS device (TSD 115) was linked to BIOPAC hardware (MP 160, BIOPAC Systems, Inc), and the responses were visualized by the experimenter through AcqKnowledge software (BIOPAC Systems, Inc, United States). In this way, when participants used the slider, the experimenter could see how much pain the participants reported. The VAS consisted of a 0- to 100-mm slider with no numerical cues, anchored with the words “no pain” (0 mm) and “the worst imaginable pain” (100 mm). The participants were instructed on the use of VAS in the following way: “*You can slide on this slider the amount of pain you felt. Bear in mind that the starting position* ‘no pain’ *means that you did not feel any pain, and that as soon as you move the slider, it means that you could discriminate the pain from other sensations*.” The same VAS and instructions were used during the experiments and the pain calibration phase.

## 3. Methods

### 3.1. Procedure: nociceptive rubber hand illusion experiment

After the written informed consent had been signed, and the handedness inventory completed, both the participant and the experimenter put on special protective glasses. Then, a laser intensity pain calibration procedure (see Supplemental Digital Content, http://links.lww.com/PAIN/C39) was conducted, followed by the N-RHI experiment.

**Figure 1.**
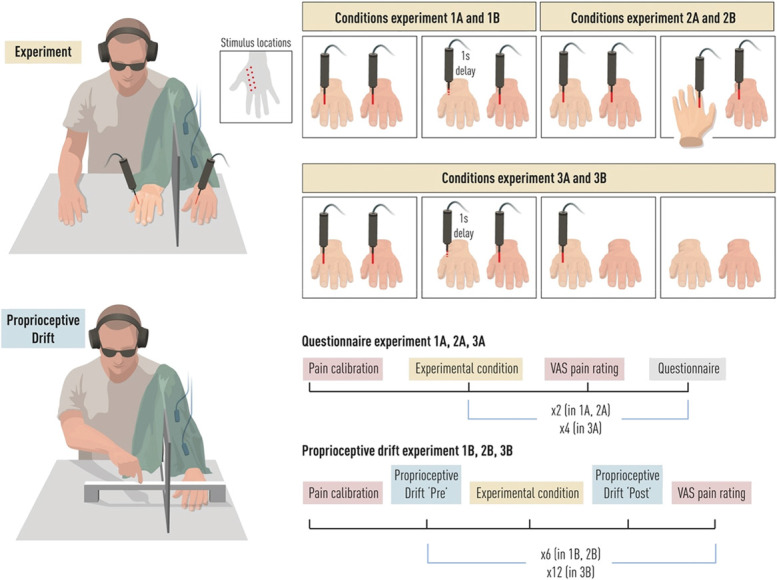
Experimental paradigm: nociceptive rubber hand illusion (N-RHI). The illustration in the top-left corner depicts the N-RHI. The diode laser and the rubber hand are in the participant's view, whereas the real hand is concealed behind a panel, out of the participant's sight. A black cloth hid the participant's shoulder and a part of the panel, completely hiding the participant's left arm and hand. The diode laser and the nociceptive laser targeted dermatomes C6/C7 of the rubber hand and the real hand (see small figure “stimulus locations”). Participants listened to low white noise through headphones and wore protective glasses during the entirety of each experiment. During experiments 3A and 3B, the EMG electrodes were placed on the skin over the left bicep and on the left extensor carpi radialis longus of the participants' left arm. The bottom left corner illustrates the procedure for the proprioceptive drift task. With their eyes closed, participants slid their right index finger toward the perceived location of their left index finger and stopped when they felt that the right index was directly above the left index. The top-right corner displays the conditions for each experiment. In Experiment 1A and Experiment 1B, the conditions were congruent (left box) and incongruent (right box). During these, synchronous diode and nociceptive laser stimuli were delivered to corresponding locations or asynchronous stimuli to nonmatching locations, respectively. In Experiment 2A and Experiment 2B, the conditions were congruent (performed as Experiments 1A and 1B; left box) and rotated (rubber hand rotated 180° from the participant's perspective; right box). In Experiment 3A and Experiment 3B, 4 conditions were used: congruent, incongruent, light, and hand (shown from left box to right box). Congruent and incongruent were the same as in Experiments 1A and 1B; in the light condition, diode laser stimulation was delivered to the rubber hand without nociceptive laser stimulation of the real hand, and in the hand condition, neither diode laser nor laser stimulation was delivered. The bottom-right corner outlines procedures for each type of experiment: questionnaire experiments (1A, 2A, 3A) and proprioceptive drift experiments (1B, 2B, 3B). Experiments 1A and 2A featured 2 conditions, each tested once in 2 separate blocks. Experiment 3A involved 4 conditions, each tested once across 4 blocks. Experiments 1B and 2B had 2 conditions, each repeated 3 times, resulting in a total of 6 blocks. Experiment 3B had 4 conditions, each tested repeated 3 times, totaling 12 blocks (3 for each condition).

The participants sat with their real left hand placed behind the screen on the table in a comfortable and relaxed position; the rubber hand was placed in full view in an anatomically plausible position to the right of the screen (Fig. 1). Participants were instructed to sit still, look at the rubber hand and not move their real hand or fingers. In Experiments 3A and 3B, we additionally monitored the muscle activity in the participant's left arm with surface electrodes (EMG, see below) placed over the left bicep and left extensor carpi radialis longus.^56^ The participants wore headphones and listened to white noise (right ear 50.7 dBA, left ear 51.5 dBA, as measured through Mini Sound Level Meter, Model ST-805, Clas Ohlson AB) so that they could not hear the “beep” made by the Nd:YAP laser machine every time a laser pulse was delivered, which otherwise might interfere with the RHI.^105^ The experimenter sat on the opposite side of the table, facing the participant.

Then, the combined nociceptive laser and visual diode laser stimulation started. During each block of the experiment, we delivered 40 nociceptive laser stimulations at a frequency of approximately 0.33 Hz on the real hand and 40 diode laser stimulations at the same frequency on the rubber hand (except in 2 of the control conditions in Experiments 3A and 3B where the nociceptive stimulation or both the visual and nociceptive stimulation were omitted). Each block lasted 120 seconds. To avoid habituation and sensitization, the laser beam was displaced to another part of dermatomes C6/C7 of the real hand by at least 5 mm. The interval between stimulations of the same skin area was at least 30 seconds. After each block of visual and nociceptive stimulation, the strength of the RHI was assessed with either the N-RHI questionnaire (Experiments 1A, 2A and 3A; see above) or the proprioceptive drift task (Experiments 1B, 2B and 3B) (see above). The questionnaire statements were delivered through cards placed in front of the participants one after the other in a completely randomized order; participants answered each statement orally, and the experimenter noted the responses on an Excel sheet. Participants rated their pain intensity felt during the last block as a retrospective average on the VAS by using a slider with their right hand, and the experimenter immediately wrote their ratings on an Excel sheet. In the experiments using a questionnaire as the outcome measure (without proprioceptive drift), 1 block was tested for each condition in line with previous RHI questionnaire experiments (2 blocks in total in Experiments 1A and 2A and 4 blocks in total in Experiment 3A).^11,35^ This ensures that the participants are as naive as possible with respect to the questionnaire items. In the proprioceptive drift experiments, each condition was tested 3 times in line with common practice (ie, to account for intratrial variability) and in line with previous studies (6 blocks in total in Experiments 2A and 2B and 12 blocks in total in Experiment 3B).^2,50,57,59^ Averages over 3 repetitions provide more accurate estimates than data from a single trial. After each block, there was a short break, when participants could relax and move their left arm and left fingers slightly to avoid muscle fatigue and to eliminate any remaining illusion experience to avoid carryover effects,^2^ before the next block commenced. The order of conditions was counterbalanced across blocks and participants; however, in Experiment 3, the fully counterbalanced design was reached after 24 participants. Therefore, we repeated the order of conditions of the first 6 participants to reach the aimed sample size. The total number of noxious stimulations in Experiments 1A, 2A, and 3A was 80, whereas that in Experiments 1B, 2B, and 3B was 240, consistent with the total number of noxious stimulations delivered in previous studies.^8^

### 3.2. Experimental conditions

In the condition used to elicit the N-RHI in all experiments (*congruent condition*), the nociceptive laser and visual diode laser stimuli were delivered synchronously and at corresponding locations on the real hand and the rubber hand.

In the *incongruent condition*, the temporal and spatial correspondences of the visual and nociceptive stimuli were violated to suppress the N-RHI. The diode laser stimuli on the rubber hand were presented with a delay of 1000 milliseconds and on a nonmatching location of the dorsum of the hand (Experiments 1A, 1B, 3A, and 3B). Instead of matching the location as in the congruent condition, we positioned the visual (on the rubber hand) and the nociceptive (on the real hand) stimuli in opposite locations, creating a spatial incongruence in addition to the temporal incongruence.

In the *rotated condition* (Experiments 2A and 2B), the rubber hand was placed in a spatially incongruent position, known to eliminate the illusion.^35,54^ Specifically, the rubber hand was rotated 180° in relation to the participant's real hand so that its fingers pointed toward the participant. Despite this rotation, the visual diode laser and nociceptive laser stimulations were delivered exactly as in the congruent condition, ie, at the same time and in the same anatomical places on the dorsum of the rubber hand and the real hand. Thus, in the rotated condition, only the visuoproprioceptive spatial incongruence was manipulated.

In Experiments 3A and 3B, alongside the incongruent condition, 2 additional control conditions were introduced that did not involve nociceptive stimulation of the real hand. In the *light condition*, participants observed the rubber hand in its usual congruent position while only the diode laser light was used to stimulate it, with no corresponding nociceptive laser stimulation on the real hand. In the *hand condition*, participants simply observed the rubber hand placed in the congruent position, without either the visual laser light or the nociceptive laser stimulation being applied. A summary of the experimental paradigm and conditions is available in Figure 1.

### 3.3. Data analysis

Data were analyzed using the statistical software RStudio 3.6.1.^104^ We set alpha at 5% (*α* = 0.05), and we used a two-tailed approach for all tests. We tested for normality through the Shapiro‒Wilk test. When data were normally distributed (ie, Shapiro‒Wilk normality test *P* value > 0.05), we used parametric tests, such as t tests; otherwise (ie, Shapiro‒Wilk normality test *P* value < 0.05), we used nonparametric analyses, such as Wilcoxon signed-rank tests. Effect sizes of the data analysis are indicated by *Cohen d*_*z*_ in relation to paired t tests and *Cohen d*_*s*_ in relation to independent sample t tests,^65^ whereas the *paired rank-biserial correlation* (*r*_*C*_) was used for nonparametric analysis^62,64^ and the *Glass biserial correlation coefficient* (*r*_*G*_)^64^ was used when nonpaired tests were run.

Although all hypotheses were directed, ie, we expected higher illusion ratings and greater proprioceptive drift in the congruent condition compared with the various control conditions, we always used 2-tailed tests. We did correct for multiple comparisons (Bonferroni–Holm correction for family wise type I errors,^48^ within each experiment, although the relevant comparisons were few (sometimes only one)) and planned a priori consistent with the previous RHI literature and our specific hypotheses; in addition, we tested the replicability of the key results in separate experiments, which further reduces the risk of false positives. When results were statistically nonsignificant, we report Bayes factor in favor of the null hypothesis (${BF}_{01}=\frac{P\left(D\;v\;H_{0}\right)}{P\left(D\;v\;H_{1}\right)}$). Bayes factors are important to support and strengthen the null finding. The Bayesian analysis was conducted using default statistical priors by the R package BayesFactor.^89^

Because we had 2 identical conditions (congruent and incongruent) in Experiments 1A and 3A and in Experiments 1B and 3B, we also conducted a post hoc analysis where we pooled the data across 2 pairs of experiments to increase the robustness of the correlation analyses; these analyses were included for purely descriptive purposes. In this larger sample, we also describe the proportion of participants who affirmed the N-RHI in the questionnaires and proprioceptive drift and explored possible correlations between the different illusion statements in line with earlier work on the classic tactile RHI,^58,107^ as well as potential correlations between the felt pain and the strength of the illusion.

## 4. Results

### 4.1. Experiment 1

#### 4.1.1. Experiment 1A

##### 4.1.1.1. Rubber hand illusion questionnaire results

**Figure 2.**
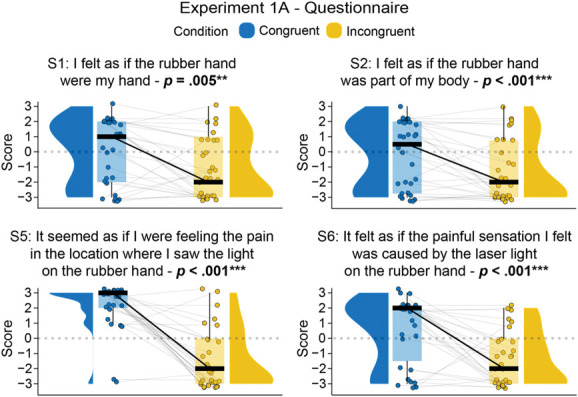
Paired comparisons in Experiment 1A: Statistically significant rubber hand illusion indicated by higher scores in illusion-related questionnaire statements when comparing the congruent versus incongruent conditions. Paired raincloud plots show individual data points and medians for each illusion-related statement in the questionnaire for Experiment 1A (N = 30). The questionnaire was administered on a Likert scale from −3 (strongly disagree) to +3 (strongly agree). **P < 0.01, ***P < 0.001, uncorrected.

In line with our hypothesis, planned comparisons revealed that ownership statements S1 and S2 were statistically significantly higher in the congruent condition than in the incongruent condition (S1: *t*_*29*_ = 3.06, *P* = 0.005, *p*_*BH*_ = 0.01, 95% CI = [0.38, 1.89], *d*_*z*_ = 0.56; S2: *V* = 149, *P* = 0.005, *p*_*BH*_ = 0.01, 95% CI = [1, 3], *r*_*C*_ = 0.74). Furthermore, the referral of pain statements S5 and S6 were also significantly higher in the congruent condition compared with the incongruent condition (S5: *V* = 345.5, *P* < 0.001, *p*_*BH*_ < 0.001, 95% CI = [3.5, 5.5], *r*_*C*_ = 0.97; S6: *t*_*29*_ = 4.35, *P* < 0.001, *p*_*BH*_ < 0.001, 95% CI = [1.02, 2.84], d_z_ = 0.79). These results are illustrated in Figure 2 and detailed in Table 3 (see also Table S1 in the Section II of the Supplemental Digital Content, http://links.lww.com/PAIN/C39).

**Table 3:** Descriptive statistics of experiment 1A and 1B.

	Measures	Congruent		Incongruent	
		Mean (±SD)	Median (1Q∼3Q)	Mean (±SD)	Median (1Q∼3Q)
Exp. 1A	S1 S2 S3 S4 S5 S6 S7 S8	0.03 (±2.08) −0.23 (±2.18) −1.63 (±1.87) −1.23 (±2.03) 2.17 (±1.58) 0.57 (±2.18) −1.2 (±1.92) −2.03 (±1.52)	1 (−2 ∼ 2) 0.5 (−2.75 ∼ 2) −3 (−3 ∼ −0.25) −2 (−3 ∼ 0) 3 (2 ∼ 3) 2 (−1.5 ∼ 2) −2 (−3 ∼ 0.75) −3 (−3 ∼ −1.25)	−1.1 (±2.01) −1.3 (±2.05) −1.57 (±1.91) −0.7 (±2.17) −1.43 (±1.96) −1.37 (±1.79) −1.63 (±1.83) −2.23 (±1.59)	−2 (−3 ∼ 1) −2 (−3 ∼ 0.75) −3 (−3 ∼ −0.25) −0.5 (−3 ∼ 1) −2 (−3 ∼ 0) −2 (−3 ∼ 0) −2.5 (−3 ∼ −1) −3 (−3 ∼ −2)
Exp. 1B	*P. Drift (mm)*	4.56 (±27.17)	0.83 (−7.5 ∼ 7.92)	−4.5 (±20.21)	−1.67 (−18.33 ∼ 5)

Descriptive statistics of the questionnaire scores in Experiment 1A and of the proprioceptive drift in experiment 1B are shown (N = 30).

Q, quartile; SD, standard deviation.

##### 4.1.1.2. Pain visual analog scale

Participants experienced similar levels of mild pain in both conditions (*t*_*29*_ = −0.82, *P* = 0.419, *p*_*BH*_ = 0.978, 95% CI = [−6.37, 2.73], *d*_*z*_ = −0.15, BF_01_ = 3.78) (Tables S2-S3 and Figure S1 in the Section II of Supplemental Digital Content, http://links.lww.com/PAIN/C39).

#### 4.1.2. Experiment 1B

##### 4.1.2.1. Proprioceptive drift results

**Figure 3.**
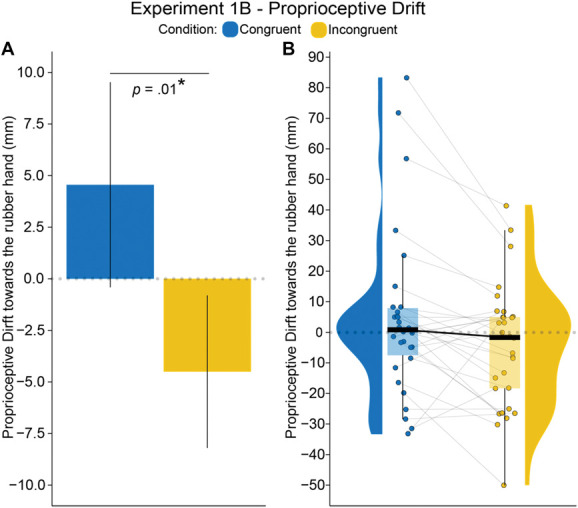
Experiment 1B plots: Statistically significant rubber hand illusion indicated by larger proprioceptive drift in congruent versus incongruent conditions. Plots show the results for the proprioceptive drift task in Experiment 1B (N = 30). (A) Bar plot with error bars showing the standard errors. (B) Paired raincloud plots show individual data points and medians. *P < 0.05.

As we had hypothesized, we observed significantly greater proprioceptive drift to the rubber hand after the congruent condition than after the incongruent condition (*t*_*29*_ = 2.72, *P* = 0.011, 95% CI = [2.26, 15.85], *d*_*z*_ = 0.5). This finding is shown in Figure 3 and Table 3 (see also Table S4 in the Section II of Supplemental Digital Content, http://links.lww.com/PAIN/C39).

##### 4.1.2.2. Pain visual analog scale

There was no difference in pain ratings between conditions, and in both conditions, similar level of mild pain was reported (*t*_*29*_ = 1.67, *P* = 0.105, *p*_*BH*_ = 1, 95% CI = [−0.31, 3.1], *d*_*z*_ = 0.31, BF_01_ = 1.49) (see Tables S5-S6 and Figure S2 in the Section II of Supplemental Digital Content, http://links.lww.com/PAIN/C39).

#### 4.1.3. Summary and interim-discussion for Experiments 1A and 1B

The questionnaire and proprioceptive drift results support that an N-RHI can be elicited by temporally and spatially congruent visual and nociceptive stimulation, thus supporting our hypothesis. More specifically, the questionnaire results showed that participants experienced both significantly stronger illusory feelings of rubber hand ownership (S1, S2) and spatial relocation of the nociceptive sensations toward the rubber hand (referral of pain, S5, S6) in the congruent condition than in the incongruent condition. The indirect behavioral proprioceptive drift measure further corroborated a stronger RHI in the congruent condition than in the incongruent control condition, showing greater spatial updating of perceived hand location toward the location of the rubber hand. Finally, we noted that the pain ratings did not vary significantly across conditions, confirming that we managed to match pain intensity across the 2 conditions and that there were no substantial sensitization or habituation effects that differed (see Tables S2, S3, S5, S6 and Figures S1, S2 in section II of Supplemental Digital Content, http://links.lww.com/PAIN/C39).

### 4.2. Experiment 2

#### 4.2.1. Experiment 2A

##### 4.2.1.1. Rubber hand illusion questionnaire results

**Figure 4.**
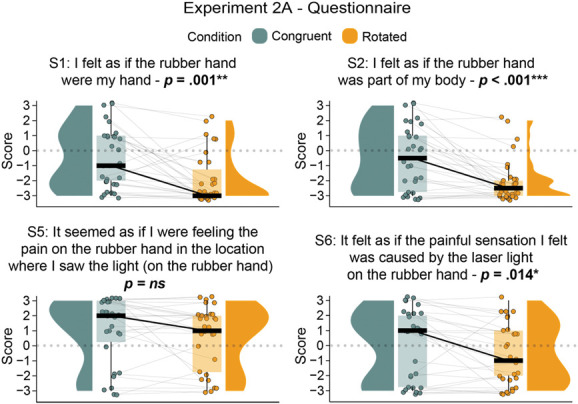
Paired comparison in Experiment 2A: Significant rubber hand illusion indicated by higher scores in illusion-related questionnaire statements when comparing congruent versus rotated conditions. Paired raincloud plots show individual data points and medians for each illusion-related statement in the questionnaire for Experiment 2A (N = 30). The questionnaire was administered on a Likert scale from −3 (strongly disagree) to +3 (strongly agree). *P < 0.05, **P < 0.01, ***P < 0.001, uncorrected.

In line with our hypothesis, planned comparison showed that the scores in the illusion-related items S1, S2, and S6 were statistically significantly higher in the congruent condition than in the rotated condition (S1: *t*_*29*_ = 3.58, *P* = 0.001, *p*_*BH*_ = 0.004, 95% CI = [0.59, 2.15], *d*_*z*_ = 0.65; S2: *V* = 186, *P* < 0.001, *p*_*BH*_ = 0.001, 95% CI = [1.5, 3.5], *r*_*C*_ = 0.96; S6: *V* = 155.5, *P* = 0.013, *p*_*BH*_ = 0.026, 95% CI = [0.5, 2], *r*_*C*_ = 0.64). However, 1 of the 2 statements related to referral of pain to the rubber hand (S5) did not show a condition-specific effect (S5: *V* = 94.5, *P* = 0.158, *p*_*BH*_ = 0.316, 95% CI = [0, 1.5], *r*_*C*_ = 0.39, BF_01_ = 1.58). These results are illustrated in Figure 4 and detailed in Table 4 (see also Table S7 in Section III of the Supplemental Digital Content, http://links.lww.com/PAIN/C39).

**Table 4:** Descriptive statistics of experiment 2A and 2B.

	Measures	Congruent		Rotated	
		Mean (±SD)	Median (1Q ∼ 3Q)	Mean (±SD)	Median (1Q ∼ 3Q)
Exp. 2A	S1 S2 S3 S4 S5 S6 S7 S8	−0.5 (±2.03) −0.47 (±2.13) −1.3 (±1.9) −0.87 (±2.06) 1.03 (±2.19) 0.07 (±2.33) −0.87 (±1.83) −2.4 (±1.33)	−1 (−2 ∼ 1) −0.5 (−2.75 ∼ 1) −2 (−3 ∼ 0) −1.5 (−2.75 ∼ 0.75) 2 (0.25 ∼ 3) 1 (−2.75 ∼ 2) −0.5 (−3 ∼ 1) −3 (−3 ∼ −2.25)	−1.87 (±1.68) −2 (±1.46) −2.47 (±1.14) −1.47 (±1.91) 0.6 (±2.11) −0.63 (±1.97) −1.17 (±1.91) −1.97 (±1.61)	−3 (−3 ∼ −1.25) −2.5 (−3 ∼ −2) −3 (−3 ∼ −2.25) −2 (−3 ∼ 0) 1 (−1.75 ∼ 2) −1 (−2 ∼ 1) −2 (−3 ∼ 0) −3 (−3 ∼ −1.25)
Exp. 2B	*P. Drift (mm)*	9.31 (±20.98)	10 (−4.58 ∼ 25.75)	−7.17 (±18.77)	−0.83 (−25.83 ∼ 6.67)

Descriptive statistics of the questionnaire scores in Experiment 2A and of the proprioceptive drift in experiment 2B are shown (N = 30).

Q, quartile; SD, standard deviation.

##### 4.2.1.2. Pain visual analog scale

There was no difference in pain ratings between the conditions (*V* = 213, *P* = 0.922, *p*_*BH*_ = 1, 95% CI = [−3.05, 2.9], *r*_*C*_ = −0.02, BF_01_ = 4.6), and the reported level of pain was mild, as indented (see Tables S8-S9 and Figure S3 in Section III of the Supplemental Digital Content, http://links.lww.com/PAIN/C39).

#### 4.2.2. Experiment 2B

##### 4.2.2.1. Proprioceptive drift results

**Figure 5.**
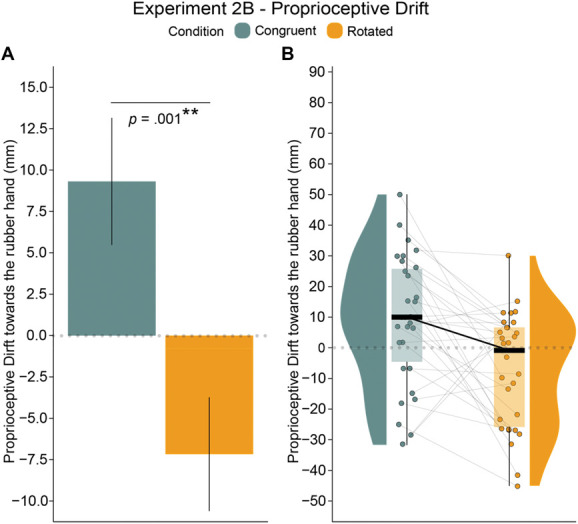
Experiment 2B: A statistically significant rubber hand illusion is indicated by larger proprioceptive drift in congruent versus rotated conditions. Plots show the results for the proprioceptive drift task in Experiment 2B (N = 30). (A) Bar plot with error bars showing the standard errors. (B) Paired raincloud plots show individual data points and medians. ** = *P* < 0.01.

The proprioceptive drift toward the rubber hand was significantly greater after the congruent condition than after the rotated condition, consistent with our hypothesis (*t*_*29*_ = 3.6, *P* = 0.001, 95% CI = [7.13, 25.83], *d*_*z*_ = 0.66) (Fig. 5 and Table 4; see also Table S10 in Section III of the Supplemental Digital Content, http://links.lww.com/PAIN/C39).

##### 4.2.2.2. Pain visual analog scale

Participants reported similar levels of mild pain after both conditions (*V* = 147.5, *P* = 0.08, *p*_*BH*_ = 0.965, 95% CI = [-3.2, 0.13], *r*_*C*_ = −0.37, BF_01_ = 0.96) (see Tables S11-S12 and Figure S4 in the Supplemental Digital Content, http://links.lww.com/PAIN/C39).

#### 4.2.3. Summary and interim-discussion for Experiments 2A and 2B

Both the questionnaire and proprioceptive drift results supported our hypothesis that placing the rubber hand in a spatially incongruent orientation with respect to the real hand—a 180-degree rotation—would eliminate the RHI, even when spatiotemporally congruent visuonociceptive stimulation was delivered. Thus, similar to the classic visuotactile RHI, the N-RHI depends on the spatial congruence of vision and proprioception. A potential limitation of the Experiments 2's questionnaire results was that the median of S1 ratings was lower than that observed in experiment 1A. The reason for this discrepancy remains unclear. However, the significantly higher S1 ratings in the congruent condition compared with the rotated condition, consistent with our hypothesis, provide evidence for the induction of the illusion. This finding aligns with the findings from other illusion-related questionnaire statements and the proprioceptive drift results in experiment 2B.

### 4.3. Experiment 3

#### 4.3.1. Experiment 3A

##### 4.3.1.1. Rubber hand illusion questionnaire results

**Figure 6.**
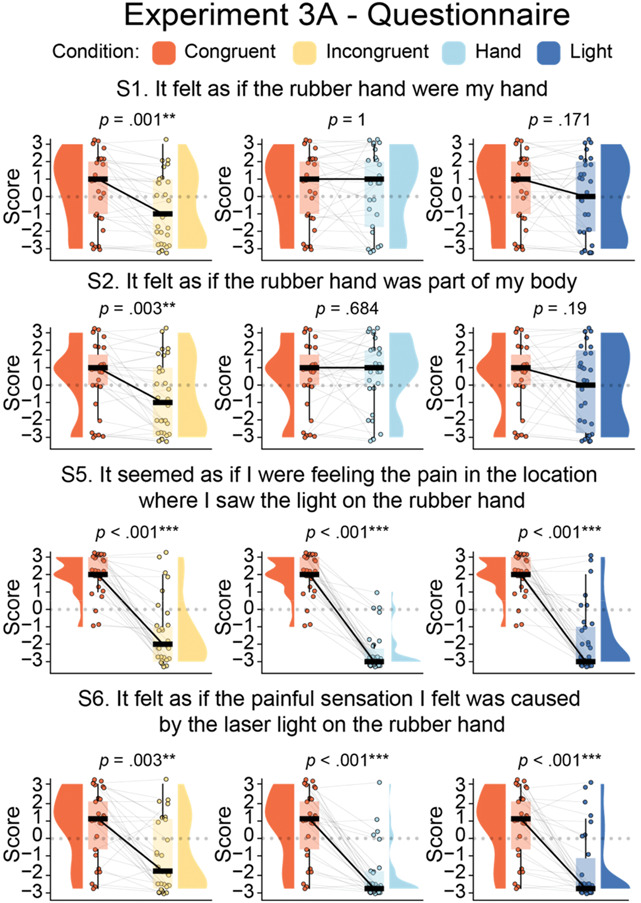
Paired comparisons in Experiment 3A: Significant rubber hand illusion indicated by higher scores in illusion-related questionnaire statements when comparing congruent versus control conditions. Paired raincloud plots show individual data points and medians for the main comparisons in Experiment 3A (N = 30). The questionnaire was administered on a Likert scale from −3 (not at all) to +3 (strongly agree). **P < 0.01, ***P < 0.001, uncorrected.

As expected, we found that the illusion-related statements S1, S2, S5, and S6 were rated significantly higher in the congruent condition than in the incongruent condition (S1: *V* = 149, *P* < 0.001, *p*_*BH*_ = 0.006, 95% CI = [1, 3], *r*_*C*_ = 0.95; S2: *V* = 201, *P* = 0.003, *p*_*BH*_ = 0.022, 95% CI = [1, 2.5], *r*_*C*_ = 0.74; S5: *V* = 325, *P* < 0.001, *p*_*BH*_ < 0.001, 95% CI = [3.5, 4.5], *r*_*C*_ = 1; S6: *t*_*29*_ = 4.6, *P* < 0.001, *p*_*BH*_ < 0.001, 95% CI = [1.07, 2.79], *d*_*z*_ = 0.84), replicating Experiment 1A. We did not find any significant difference in ownership statements S1 and S2 when comparing the congruent condition against the hand condition (S1: *V* = 86, *P* = 0.982, *p*_*BH*_ = 1, 95% CI = [−1.5, 1], *r*_*C*_ = 0.01, BF_01_ = 5.14; S2: *V* = 103.5, *P* = 0.671, *p*_*BH*_ = 1, 95% CI = [−1.5, 1], *r*_*C*_ = −0.1, BF_01_ = 4.87). We did not find any difference in illusion statements S1 and S2, which relate to feelings of hand ownership, when comparing the congruent condition with the light condition (S1: *t* = 1.4, *P* = 0.171, *p*_*BH*_ = 0.785, 95% CI = [−0.24, 1.31], *d*_*z*_ = 0.26, BF_01_ = 2.12; S2: *V* = 104.5, *P* = 0.182, *p*_*BH*_ = 0.785, 95% CI = [−0.5, 3], *r*_*C*_ = 0.37, BF_01_ = 2.12). We also found that in the incongruent condition, participants reported less ownership sensation of the rubber hand compared with the hand condition (S1: *V* = 192, *P* < 0.001, *p*_*BH*_ = 0.01, 95% CI = [1, 2.5], *r*_*C*_ = 0.83; S2: *V* = 171.5, *P* = 0.002, *p*_*BH*_ = 0.019, 95% CI = [1, 3], *r*_*C*_ = 0.81) (Fig. 6 and Table 5; see Tables S13 in Section IV of the Supplemental Digital Content, http://links.lww.com/PAIN/C39).

**Table 5:** Descriptive statistics of experiment 3A and 3B.

	Measures	Congruent		Incongruent		Hand		Light	
		Mean (±SD)	Median (1Q ∼ 3Q)	Mean (±SD)	Median (1Q ∼ 3Q)	Mean (±SD)	Median (1Q ∼ 3Q)	Mean (±SD)	Median (1Q ∼ 3Q)
Exp. 3A	S1 S2 S3 S4 S5 S6 S7 S8	0.4 (±2.06) 0.47 (±1.96) −1.23 (±1.85) −1.07 (±2.16) 1.97 (±1.13) 0.73 (±1.98) −1.07 (±1.86) −1.63 (±1.88)	1 (−1 ∼ 2) 1 (0 ∼ 1.75) −2 (−3 ∼ 0.75) −1.5 (−3 ∼ 1) 2 (2 ∼ 3) 1 (−0.75 ∼ 2) −2 (−3 ∼ 0.75) −3 (−3 ∼ −0.25)	−0.8 (±1.99) −0.7 (±2.12) −1.33 (±1.86) −0.93 (±1.86) −1.33 (±1.86) −1.2 (±2.02) −1.27 (±1.78) −1.8 (±1.81)	−1 (−3 ∼ 1) −1 (−3 ∼ 1) −2 (−3 ∼ 0) −1 (−3 ∼ 0.75) −2 (−3 ∼ −1) −2 (−3 ∼ 1) −2 (−3 ∼ 0.5) −3 (−3 ∼ −0.5)	0.4 (±2.24) 0.6 (±2.03) −1.1 (±1.97) −0.93 (±1.98) −2.43 (±1.14) −2.23 (±1.5) −2.27 (±1.41) −1.27 (±2.18)	1 (−1.75 ∼ 2) 1 (0 ∼ 2) −2 (−3 ∼ 0.75) −1.5 (−3 ∼ 1) −3 (−3 ∼ −2.25) −3 (−3 ∼ −2) −3 (−3 ∼ −2) −2.5 (−3 ∼ 1)	−0.13 (±2.21) −0.13 (±2.29) −1.53 (±1.72) −1.03 (±2.16) −1.7 (±1.86) −1.87 (±1.98) −2.6 (±0.97) −0.77 (±2.31)	0 (−2 ∼ 2) 0 (−2.75 ∼ 2) −2 (−3 ∼ −0.25) −2 (−3 ∼ 1) −3 (−3 ∼ −1) −3 (−3 ∼ −1.25) −3 (−3 ∼ −2.25) −2 (−3 ∼ 1)
Exp. 3B	*P. Drift (mm)*	16.9 (±22.4)	13.3 (2.1 ∼ 29.6)	4.7 (±18.9)	8.3 (−9.6 ∼ 18.3)	7.2 (±22.4)	5.8 (−6.2 ∼ 13.3)	5.6 (±18.4)	5 (−9.2 ∼ 17.9)

Descriptive statistics of the questionnaire scores in Experiment 3A and of the proprioceptive drift in experiment 3B are shown (N = 30).

Q, quartile; SD, standard deviation.

##### 4.3.1.2. Pain visual analog scale

There was no difference in pain ratings between congruent and incongruent conditions (*t*_*29*_ = 0.82, *P* = 0.417, *p*_*BH*_ = 1, 95% CI = [−1.37, 3.22], *d*_*z*_ = 0.15, BF_01_ = 3.77) (see Tables S14-S15 and Figure S5 in Section IV of the Supplemental Digital Content, http://links.lww.com/PAIN/C39).

##### 4.3.1.3. Electromyography results

The average (120 seconds of the block) EMG activity did not differ between the congruent and incongruent blocks (extensor: *V* = 267, *P* = 0.289, *p*_*BH*_ = 1, 95% CI = [0, 0], *r*_*C*_ = 0.23, BF_01_ = 3.17; bicep: *V* = 279, *P* = 0.187, *p*_*BH*_ = 1, 95% CI = [0, 0], *r*_*C*_ = 0.28, BF_01_ = 2.73). The block-average EMG activity was not significantly different between the congruent and light conditions (extensor: *t*_*28*_ = 0.52, *P* = 0.604, *p*_*BH*_ = 1, 95% CI = [0, 0], *d*_*z*_ = 0.1, BF_01_ = 4.46; bicep: *t*_*28*_ = 0.65, *P* = 0.522, *p*_*BH*_ = 1, 95% CI = [0, 0], *d*_*z*_ = 0.12, BF_01_ = 4.17). Similarly, there was no significant difference in the average EMG activity throughout the block between any other conditions for either the left bicep muscle or the left-hand extensor muscle. In addition, there were no differences of the averages of the muscular activity in the 200-millisecond window after each laser stimulation between the congruent and incongruent conditions for either the left extensor (*V* = 236, *P* = 0.697, *p*_*BH*_ = 1, 95% CI = [0, 0], *r*_*C*_ = 0.09, BF_01_ = 4.57) or left bicep muscle (*V* = 276, *P* = 0.21, *p*_*BH*_ = 1, 95% CI = [0, 0], *r*_*C*_ = 0.27, BF_01_ = 2.55) (see Tables S16-S17 and Figure S6 in Section IV of the Supplemental Digital Content, http://links.lww.com/PAIN/C39). This verifies that the participants' left arms and hands were similarly relaxed across conditions as instructed and that the laser stimulation did not produce involuntary muscular contractions or twitches.

#### 4.3.2. Experiment 3B

##### 4.3.2.1. Proprioceptive drift results

**Figure 7.**
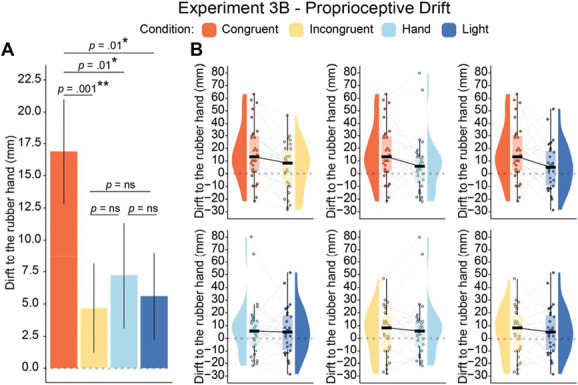
Experiment 3B: Significant rubber hand illusion indicated by larger proprioceptive drift in congruent versus control conditions. Plots showing the results for the proprioceptive drift task in Experiment 3B (N = 30). (A) Bar plot with error bars showing the standard errors. (B) Paired raincloud plots show individual data points and medians. *P < 0.05, **P < 0.01, uncorrected.

The 3 planned comparisons revealed that the proprioceptive drift was significantly greater after the congruent condition compared with each of the 3 control conditions in line with our a priori hypothesis: incongruent condition (*t*_*29*_ = 3.06, *P* = 0.005, *p*_*BH*_ = 0.028, 95% CI = [4.06, 20.38], *d*_*z*_ = 0.56), hand condition (*t*_*29*_ = 2.6, *P* = 0.015, *p*_*BH*_ = 0.073, 95% CI = [2.05, 17.28], *d*_*z*_ = 0.47), and light condition (*t*_*29*_ = 2.58, *P* = 0.015, *p*_*BH*_ = 0.073, 95% CI = [2.33, 20.23], *d*_*z*_ = 0.47) (Fig. 7 and Table 5, see also Table S18 in Section IV of the Supplemental Digital Content, http://links.lww.com/PAIN/C39).

##### 4.3.2.2. Pain visual analog scale

There was no perceived difference in pain ratings between the congruent and incongruent conditions, which in both cases was mild as intended (*V* = 284.5, *P* = 0.285, *p*_*BH*_ = 1, 95% CI = [−1.12, 2.81], *r*_*C*_ = 0.22, BF_01_ = 4.99) (see Tables S19-S20 and Figure S7 in Section IV of the Supplemental Digital Content, http://links.lww.com/PAIN/C39*)*.

##### 4.3.2.3. Electromyography results

The average EMG activity from the 120-second block (block-average EMG) did not differ between the congruent and incongruent blocks (extensor: *V* = 257, *P* = 0.393, *p*_*BH*_ = 1, 95% CI = [0, 0], *r*_*C*_ = 0.18, BF_01_ = 3.11; bicep: *V* = 255, *P* = 0.417, *p*_*BH*_ = 1, 95% CI = [0, 0], *r*_*C*_ = 0.17, BF_01_ = 3.25). The block-average EMG activity was significantly lower in the congruent condition than in the hand condition (extensor: *t*_*28*_ = −2.36, *P* = 0.026, *p*_*BH*_ = 0.307, 95% CI = [0, 0], *d*_*z*_ = −0.44; bicep: *t*_*28*_ = −2.34, *P* = 0.026, *p*_*BH*_ = 0.307, 95% CI = [0, 0], *d*_*z*_ = −0.44), but there was no significant difference between the congruent and light conditions (extensor: *t*_*28*_ = −0.13, *P* = 0.899, *p*_*BH*_ = 1, 95% CI = [0, 0], *d*_*z*_ = −0.02, BF_01_ = 5.03; bicep: *t*_*28*_ = −0.29, *P* = 0.775, *p*_*BH*_ = 1, 95% CI = [0, 0], *d*_*z*_ = −0.05, BF_01_ = 4.88). However, the block-averaged EMG activity was significantly higher in the hand condition than in the incongruent condition (extensor: *t*_*28*_ = 2.92, *P* = 0.007, *p*_*BH*_ = 0.094, 95% CI = [0, 0], *d*_*z*_ = 0.54; bicep: *t*_*28*_ = 2.93, *P* = 0.007, *p*_*BH*_ = 0.094, 95% CI = [0, 0], *d*_*z*_ = 0.54) and the light condition (extensor: *t*_*28*_ = 2.31, *P* = 0.029, *p*_*BH*_ = 0.307, 95% CI = [0, 0], *d*_*z*_ = 0.43; bicep: *t*_*28*_ = 2.19, *P* = 0.037, *p*_*BH*_ = 0.33, 95% CI = [0, 0.0], *d*_*z*_ = 0.41).

No differences between the congruent and incongruent conditions were found when the laser-evoked EMG (average of the 200-ms window after each laser input) was analyzed for either the left extensor (*V* = 258, *P* = 0.381, *p*_*BH*_ = 1, 95% CI = [−0, 0], *r*_*C*_ = 0.19, BF_01_ = 3.02) or the left bicep (*V* = 254, *P* = 0.43, *p*_*BH*_ = 1, 95% CI = [−0, 0], *r*_*C*_ = 0.17, BF_01_ = 3.17) (see Tables S21-S22 and Figure S8 in the Supplemental Digital Content, http://links.lww.com/PAIN/C39).

#### 4.3.3. Summary and interim-discussion for Experiments 3A and 3B

The questionnaire and proprioceptive drift outcomes from the congruent and incongruent conditions replicated the findings from Experiments 1A and 1B. Once again, congruent visuonociceptive stimulation led to a significantly stronger N-RHI reports. Moreover, the proprioceptive drift data supported our hypothesis of stronger RHI in the congruent condition compared with the incongruent condition and the 2 additional conditions where no nociceptive stimulation was delivered (light and hand conditions). Thus, the results are overall consistent with our hypothesis regarding the N-RHI.

However, the hand ownership ratings in the congruent illusion condition of Experiment 3A did not significantly surpass those in the hand and light control conditions. Nonetheless, the implications of these nonsignificant observations remain ambiguous. The absence of a notable proprioceptive drift in these control conditions suggests a lack of a robust RHI. Although simply observing a rubber hand may induce a subjective illusion due to visuoproprioceptive integration in some instances,^57,108^ this influence should also extend to the proprioceptive drift. Furthermore, these controls were not as closely matched to the congruent condition as the incongruent condition was, primarily because they lacked nociceptive stimulation. This discrepancy suggests that the subjective ratings might have been influenced by unspecific cognitive effects related to the pain experience or the lack thereof. This could potentially cause participants to focus more on the real hand and less on the rubber hand in conditions when pain was delivered and focus more on the rubber hand and less on the real hand in the hand and light conditions. This may, in turn, have influenced the illusion reports.^118^ Therefore, our primary conclusions from Experiments 3A and 3B are based on the differences in subjective illusion between the well-matched congruent and incongruent conditions, as well as the observation of significant differences in proprioceptive drift between the congruent condition and all control conditions, including the hand and light conditions.

### 4.4. Post hoc analysis: pooling data across experiments

Data from questionnaire-based experiments 1A and 3A, as well as from proprioceptive drift experiments 1B and 3B, were combined to examine the robustness of the findings. This combination allowed us to assess the proportion of participants who affirmed the N-RHI in the questionnaire, demonstrated an illusion-related proprioceptive drift effect, and to explore potential correlations between individual illusion questionnaire statements. We also investigated correlations between the illusion measures (questionnaire statements and proprioceptive drift) and the VAS ratings. By combining the data from 2 experiments, we obtained a sample size (n = 60) that is more suitable for exploring correlations (see Tables S23 and S29 in Section V of the Supplemental Digital Content, http://links.lww.com/PAIN/C39). It is important to note that these experiments included both congruent and incongruent conditions, involving 2 different groups of participants; thus, the data could be appropriately pooled.

The results from the questionnaire and proprioceptive drift mirrored those from the individual experiments (see Tables S24, S25, S30, S31, and Figure S9 and S12 in Section V of the Supplemental Digital Content, http://links.lww.com/PAIN/C39). In addition, we noted that 55% (n = 33) of the total sample (n = 60) experienced the N-RHI, defining an illusion responder as one with an S1 score of ≥ 1. In a similar vein, 70% (n = 42) of the total sample exhibited a more pronounced proprioceptive drift in the congruent condition compared with the incongruent condition.

Next, we explored potential correlations between statements of ownership (S1 and S2) and the referral of pain (S5 and S6). This is analogous to previously described correlations between ownership and the referral of touch in the classic visuotactile RHI.^107^ We conducted correlational analyses on the difference scores derived by subtracting the scores of the incongruent condition from the congruent condition. Significant positive correlations emerged between ownership statement S1 and referral of pain statement S5 (*r*_*S*_ = 0.33, *P* = 0.01, *p*_*BH*_ = 0.177), between ownership statement S1 and referral of pain statement S6 (*r*_*S*_ = 0.67, *P* < 0.001, *p*_*BH*_ < 0.001), and between ownership statement S2 and referral of pain statement S6 (*r*_*S*_ = 0.44, *P* < 0.001, *p*_*BH*_ = 0.007). However, no significant correlation was found between ownership statement S2 and referral of pain statement S5 (*r*_*S*_ = 0.19, *P* = 0.14, *p*_*BH*_ = 1, BF_01_ = 1.22). Further details can be found in Tables S28 and Figure S11 of Section V of the Supplemental Digital Content, http://links.lww.com/PAIN/C39. These findings suggest that the sensation of pain emanating from the rubber hand and the perception of the rubber hand being one's own are interrelated in the N-RHI.

Finally, we explored the correlations between illusion ratings and pain VAS ratings, and proprioceptive drift and pain VAS ratings. No significant correlation was found between ownership statements and pain VAS ratings neither in the congruent condition (S1-VAS: *r*_*S*_ = −0.05, *P* = 0.685, *p*_*BH*_ = 1, BF_01_ = 3.04; S2: *r*_*S*_ = −0.05, *P* = 0.715, *p*_*BH*_ = 1, BF_01_ = 3.32) nor in the incongruent condition (S1-VAS: *r*_*S*_ = −0.07, *P* = 0.618, *p*_*BH*_ = 1, BF_01_ = 2.47; S2: *r*_*S*_ = −0.15, *P* = 0.243, *p*_*BH*_ = 1, BF_01_ = 3.42) (see Table S28 and Figure S11 in Section V of the Supplemental Digital Content, http://links.lww.com/PAIN/C39).

In the congruent condition, there was no significant correlation between proprioceptive drift and rated pain intensity (*r*_*S*_ = 0.06, *P* = 0.647, *p*_*BH*_ = 1, BF_01_ = 0.81). On the other hand, in the incongruent condition, a positive correlation was observed between proprioceptive drift and VAS pain ratings. However, this correlation did not remain significant after correction for multiple comparisons, making its relevance unclear (*r*_*S*_ = 0.26, *P* = 0.045, *p*_*BH*_ = 0.179) (see Table S34 and Figure S14 in Section V of the Supplemental Digital Content, http://links.lww.com/PAIN/C39).

## 5. Discussion

In this study, we investigated whether contactless Nd:YAP laser stimulation of nociceptive afferents in the skin (C fibers and Aδ fibers) can elicit the RHI when presented with congruent visual cues. Significant and replicable questionnaire-based and proprioceptive drift results from 6 experiments supported that the RHI could be elicited using nociceptive instead of tactile stimuli. Importantly, the illusion was only elicited when the nociceptive signals from the participants' hidden real hand and the visual signals from the rubber hand in view were spatiotemporally matched, which suggests that similar multisensory integration principles determine the RHI triggered by selective nociceptive stimulation as those in the classic variant of the illusion triggered by tactile stimulation of the skin. These observations suggest that information from C fibers and Aδ fibers can integrate with visual and proprioceptive information from the body and lead to changes in bodily awareness and the feeling of a limb as one's own.

This observation has important conceptual implications for both body representation research and cognitive pain research. As mentioned, only 2 studies^19,23^ have investigated the role of painful stimuli in the RHI, but these studies did not use stimulation protocols that allowed the selective stimulation of nociceptive fibers (ie, C fibers and Aδ fibers) as in the current contactless laser–based approach, which means that the contribution of tactile signals could not be excluded in these previous studies. Thus, the current results expand our understanding of the types of sensory systems that contribute to the sense of body ownership by providing conclusive evidence for a role of nociceptive information. This has implications for models of body ownership, which should consider the role of signals from nociceptive C fibers and Aδ fibers, either as an additional source of sensory information that determines the combination or segregation of bodily signals, as in Bayesian models of body ownership,^22,38,108^ or as nociceptive predictions and nociceptive prediction errors, as in predictive coding and free-energy formulations of body ownership.^4^ Moreover, the current results are relevant for models of body ownership that emphasize the role of interoception^97,103,125^ and inputs from C fibers in the skin,^29,30,121^ as signals from C fibers provide an important source of information about the physiological state of the body^26^ from both inner organs and the skin.^26,29^

For cognitive pain research, the observation that nociceptive pain^87^ information is integrated into a multisensory representation of one's own body is relevant, as it implies that feeling pain from a particular part of the body is the result of a complex integration process where visual, tactile, proprioceptive, and nociceptive signals are combined to produce a coherent multisensory experience of one's own body part in pain. This view is different from neuroscience and medical textbooks that emphasize the *unisensory* processing of nociceptive information and explain the localization of the resulting pain on the body as simply resulting from activation of different somatotopically organized representations in the somatosensory cortex^61^ and the activation of central nociceptive and pain processing pathways (eg, insular and anterior cingulate cortex).^55,61,69,84,85,100^ However, nociceptive information is “meaningless” unless integrated with information from other bodily senses and incorporated into a central body representation so that the painful sensation becomes part of how one experiences the body and how appropriate behavioral protective defense reactions can be generated.^68,69,91^ This integration of nociceptive pain information with tactile, proprioceptive, and visual information and other bodily senses into a coherent multisensory representation of a hurting limb presumably involves multisensory areas in the frontal and parietal association cortex,^35,45^ although future neuroimaging studies are needed to test this hypothesis at the neural level. This integrative embodied perspective on nociceptive pain is relevant for research into the interplay between body representation and pain processing in pain disorders where changes in bodily awareness and pain often co-occur, such as phantom limb pain,^32,42^ complex regional pain syndrome,^71,82,90^ fibromyalgia,^83,111^ chronic spinal cord injuries,^110^ and chronic lower back pain,^74,94,122^ and for research into the mechanisms of mirror therapy and virtual reality–based bodily illusion therapy^20,86,106,112,122^ that aims to alleviate pain through manipulation of bodily multisensory integration mechanisms (eg, phantom limb pain).^96^

The current results also advance our understanding of the remapping of nociceptive pain signals from a somatotopic spatial reference system (“skin coordinates”) to an external spatial reference system (space near the body). The RHI requires the combination of visual and somatosensory information that is initially coded in different spatial reference frames (visual information in retinotopic space and somatosensory information in somatotopic space); to enable effective integration, the signals are remapped into a common spatial reference frame^7,47,120^ (although the remapping can be partial, see 6). Body part–centered spatial reference frames in space near the body (peripersonal space) provide a common coordinate system for visuotactile integration,^18,43,44^ and such spatial remapping is used in the rubber hand illusion.^17,98,133^ Thus, the current findings suggest that nociceptive pain sensations are also remapped into a common external coordinate system in the peripersonal space surrounding the upper limb during nociceptive RHI. Further evidence for this in our data was the observation that during the illusion, our volunteers reported that the pain they felt was located on the rubber hand (S5) and was caused by the diode laser light shining on the fake hand (S6).

This finding is consistent with previous studies^109,129^ that used a reaction time–based nociceptive stimulus simultaneity task. The task was based on a classic tactile simultaneity task used to investigate spatial remapping of touch. During the task, participants held their hands in either a crossed or uncrossed posture and judged whether tactile stimulations delivered to both hands occurred simultaneously. The task became more challenging when the hands were crossed.^46,114^ The observed changes in reaction time during this task when using painful stimuli suggest that painful signals are also remapped into a common external spatial coordinate system, similar to tactile signals. Remapping painful information in peripersonal space might not only serve the flexibility of localizing and identifying one's limbs but may also enhance the detection of physical threats near the body,^69^ which would be consistent with studies that found that physically threatening the rubber hand during the RHI led to enhanced emotional and psychophysiological defense reactions.^5,36,37,41^

Although our study was not designed to investigate how variations in the intensity of pain might modulate the rubber hand illusion or how the strength of the rubber hand illusion might modulate the experience of pain (we did our best to keep pain VAS ratings similarly low in all subjects and conditions), we did not observe any correlation between pain ratings and the subjective ratings of the illusion or the proprioceptive drift measure. Studies have investigated the potential “analgesic” effect of the rubber hand illusion,^75,76,79^ ie, the idea that the rubber hand illusion might modulate pain thresholds, although the findings are mixed. The current data do not support such an analgesic effect, consistent with a previous study.^88^ This lack of correlation also disputes the possibility that pain, being an alarming signal for potential tissue damage, might have “interrupted” the illusion by forcing attention toward the hidden real hand. Furthermore, our EMG recordings ruled out the putative concern that the nociceptive stimulation might have triggered small involuntary movements or static muscular contractions from the real arm, which might have interrupted the illusion by providing incongruent proprioceptive sensory feedback.

A couple of limitations of the study should be acknowledged. First, the average scores on the ownership statements were slightly lower compared with those in the classical RHI experiments^5,43,56^; although the proprioceptive drift effect was in line with previous RHI studies.^30,57,58,70,105^ One reason for the relatively lower RHI ratings may be that in the current paradigm, the nociceptive stimuli were much shorter in duration (7 ms, although they were perceived for longer) than the tactile stimulations with brushstroke stimuli (typically 0.5-1 second). Thus, the brief nociceptive and visual laser stimuli we used probably contained less sensory information than the classic tactile RHI stimulation, which, according to the probabilistic models of the RHI, would lead to a weaker illusion.^22,108^ Another possibility is that nociception is weighted lower than touch in the integration of bodily signals and therefore might contribute less to the illusion; differences in the relative weighting of different modalities have been noted before in bodily illusion experiments.^101^ Second, in Experiment 3A's congruent illusion condition, hand ownership ratings were not significantly different from the hand and light control conditions. However, the implications of these results are unclear, as no noteworthy changes in proprioceptive drift were observed in these control conditions, indicating a weak RHI. In addition, unlike in the congruent and incongruent conditions, these additional controls lacked nociceptive stimulation—a difference that may have influenced the illusion reports due to nonspecific cognitive effects, such as directing more attention to the rubber hand than to the real hand. Thus, we primarily draw our conclusions from the consistent subjective illusion differences between the well-matched congruent and incongruent conditions, and the significant proprioceptive drift differences between the congruent condition and all controls across all experiments. Finally, this study used contactless laser stimulation to stimulate nociceptive C fibers and Aδ fibers. This raises the question of whether stimulation of *non*nociceptive C fibers using this technique, such as thermosensory C-fibers, can also lead to changes in illusory body ownership, which remains open for future investigations.

In conclusion, pain not only serves to detect tissue damage and support the emotional experience of pain but also contributes to the sense of the body as one's own. The fact that nociceptive information seamlessly integrates with sensory signals from other modalities in multisensory bodily awareness opens up new horizons in the study of nociceptive pain as an embodied sensory experience.

## Conflict of interest statement

The authors have no conflicts of interest to declare.

## Appendix A. Supplemental digital content

Supplemental digital content associated with this article can be found online at http://links.lww.com/PAIN/C39.

## Supplemental video content

A video abstract associated with this article can be found on the PAIN Web site.

## Supplementary Material

SUPPLEMENTARY MATERIAL
