# Supplementary material for: Eliciting the rubber hand illusion by the activation of nociceptive C and Aδ fibers
Source: Pain. 2024 May 24;165(10):2240–56. doi: 10.1097/j.pain.0000000000003245 (PMC11404332; doi:10.1097/j.pain.0000000000003245)
Supplement: SUPPLEMENTARY MATERIAL [file jop-165-2240-s001.pdf]

Supplemental digital content

Eliciting the rubber hand illusion by the activation of nociceptive C and Aδ fibers

Sara Coppi[1], Karin Jensen[2], H. Henrik Ehrsson[1]

[1]Department of Neuroscience, Karolinska Institutet, Stockholm, Sweden.
[2]Department of Clinical Neuroscience, Karolinska Institutet, Stockholm, Sweden.

Table of Content

Section I – METHODS…………………………………………………………………………...p. 4

Section II – Experiment 1………………………………………………...……………………p. 6
(Tables S1 – S6, Figures S1 – S2)

Table S1 – *Analysis questionnaire, experiment 1A*
Table S2 – *VAS Descriptive statistics, experiment 1A*
Table S3 – *Analysis VAS, experiment 1A*
Figure S1 – *Raincloud plots VAS data, experiment 1A*

Table S4 – *Analysis proprioceptive drift, experiment 1B*
Table S5 – *VAS Descriptive statistics, experiment 1B*
Table S6 – *Analysis VAS, experiment 1B*
Figure S2 – *Raincloud plots in the VAS, Experiment 1B*

Section III – Experiment 2……………………………………………………….………………p. 12
(Tables S7 – S12, Figures S3 – S12)

Table S7 – *Analysis questionnaire, experiment 2A*
Table S8 – *VAS Descriptive statistics, experiment 2A*
Table S9 – *Analysis VAS, experiment 2A*
Figure S3 – *Raincloud plots VAS data, Experiment 2A*

Table S10 – *Analysis proprioceptive drift, experiment 2B*
Table S11 – *VAS Descriptive statistics, experiment 2B*
Table S12 – *Analysis VAS, experiment 2B*
Figure S4 – *Raincloud plots for VAS data, Experiment 2B*

Section IV – Experiment 3……………………………………………………………….…p. 18
(Tables S13 – S22, Figures S5 – S8)

Table S13 – *Analysis questionnaire, experiment 3A*
Table S14 – *VAS Descriptive statistics, experiment 3A*
Table S15 – *Analysis VAS, experiment 3A*
Figure S5 – *Raincloud plots VAS data, Experiment 3A*

Table S16 – *EMG Descriptive statistics, experiment 3A*
Table S17 – *Analysis EMG, experiment 3A*
Figure S6 – *Bar plots EMG data, experiment 3A*

Table S18 – *Analysis proprioceptive drift, experiment 3B*
Table S19 – *VAS Descriptive statistics, experiment 3B*
Table S20 – *Analysis VAS, experiment 3B*
Figure S7 – *Raincloud plots for VAS data, Experiment 3B*

Table S21 – *EMG Descriptive statistics, experiment 3B*
Table S22 – *Analysis EMG, experiment 3B*
Figure S8 – *Bar plots EMG data, experiment 3B*

Section V – Post-Hoc Pooled Analysis……………………………………………...………p. 28

(Tables S23 – S34, Figures S9 – S14)

Table S23 – *Descriptive statistics participants, experiments 1A+3A*
Table S24 – *Descriptive statistics questionnaire, experiments 1A+3A*
Table S25 – *Analysis questionnaire, experiments 1A+3A*
Figure S9 – *Raincloud plots questionnaire, experiments 1A+3A*

Table S26 – *VAS Descriptive statistics, experiments 1A+3A*
Table S27 – *Analysis VAS, experiments 1A+3A*
Figure S10 – *Raincloud plots for VAS data, experiments 1A+3A*

Table S28 – *Correlational analyses between questionnaire and VAS, experiments 1A+3A*
Figure S11 – *Correlational plots, experiments 1A+3A*

Table S29 – *Descriptive statistics participants, experiments 1B+3B*
Table S30 – *Descriptive statistics proprioceptive drift, experiments 1B+3B*
Table S31 – *Analysis proprioceptive drift, experiments 1B+3B*
Figure S12 – *Plots proprioceptive drift, experiments 1B+3B*

Table S32 – *VAS Descriptive statistics, experiments 1B+3B*
Table S33 – *Analysis VAS, experiments 1B+3B*
Figure S13 – *Raincloud plots for VAS data, experiments 1B+3B*

Table S34 – *Correlational analyses between proprioceptive drift and VAS, experiments 1B+3B*
Figure S14 – *Correlational plots, experiments 1B+3B*

Bibliography…………………………………………………………………………………...…… p. 41

SECTION I – METHODS

Calibration of the nociceptive laser intensity and subjective pain

The painful laser stimulations used in this study were perception locked. Before each N-RHI experiment, we calibrated the intensity of the laser stimulation for each participant to select the appropriate level of energy fluence to elicit mild pain and to match the level of subjectively experienced pain across participants. The aim of the calibration phase was to ensure nociceptive activation according to the subjective pain report as well as report descriptors such as 'hot/burning' and/or 'pinpricking'. The intensity of the laser was based on the participant's sensitivity and assessed through a staircase procedure (see below). We aimed to reach a mild pain sensation, between 1 and 40 on the VAS. The calibration procedure for Experiments 1A and 1B was as follows: The participant was seated comfortably in front of a desk and placed their left arm on the desk behind an occluding screen. The experimenter started by delivering one laser stimulus with the smallest amount of energy (i.e., 1 Joule) and increased it by 0.25 Joules for each new stimulus. The new stimulus was not delivered in the same place as the previous stimulus but remained within dermatomes C6 and C7. The calibration procedure included a staircase method with increasing and decreasing scales of energy as follows: if after the first stimulus the participant did not feel anything, the energy was increased by 0.5 J; if the participant started to feel something (usually a warm sensation), it was increased by 0.25 J. After each step, the experimenter asked the following questions: "Did you feel any pain?" and "Did you feel any sensation? If so, what type of sensation?". For the first question, the participant needed to answer either yes or no, and for the second, they could freely report what was felt. If the participant felt pain, they were asked to indicate on the slider how much pain they felt (see above). When the participant started to feel pain, the experimenter increased the energy by 0.25 J and started a decreasing scale. During this procedure, the participant needed to state when they no longer felt pain. When the participant no longer felt pain, the energy was increased one step further, and the experimenter delivered another stimulus. If the subject felt pain, then that amount of energy was set for the experiment. If the subject did not feel pain, the experimenter decreased the energy by one step and started the increasing scale again. The final energy was the one that caused the participant to start to feel pain. Each stimulus used in the staircase procedure was delivered approximately once every 10 seconds. After the ascending/descending pain calibration procedure, the experimenter asked whether the painful stimulation elicited sensations of 'hot/burning' or 'pinpricking'.

For Experiments 2A to 3B, we used a modified version of the above pain intensity calibration procedure. Instead of using only one laser pulse in the staircase procedures, we presented a series of laser stimulations (starting from 1 and reaching 20, in steps of 5). These trains of stimuli were delivered, as in the experiments, every three seconds (~ 0.33 Hz) and to different areas (displaced by at least 5 mm) of the dorsum of the left hand. Additionally, in the new procedure, the stimulated hand was in the participant's view. The change was motivated by the fact that we wanted the pain calibration to be as similar as possible to the trains of nociceptive stimulations given in the N-RHI conditions. The pain calibration results for each experiment are shown in Table 2. Upon completion of the calibration phase, the energy for the laser pulses used in the experiments was confirmed to consistently elicit a distinct 'hot/burning' sensation in all participants (n = 180) and a clear 'pinpricking' sensation in 91% of the participants (n = 164).

Statistical Analysis

All the statistical analyses reported here follow the same methods explained in the main manuscript (paragraph 3.5). However here, we also report all the analyses that are not reported in the main manuscript. Additionally, here we report the Bayes Factors (BF) (i.e., $BF_{10} = \frac{P(D|H_1)}{P(D|H_0)}$ or $BF_{01} = \frac{P(D|H_0)}{P(D|H_1)}$). The Bayesian analysis was conducted using default statistical priors by the R package BayesFactor [83]. BF_{10} was reported when there was evidence in support of the alternative hypothesis; conversely, BF_{01} was reported when evidence was in favour of the null hypothesis.

VAS

Methods

During the experiments, subjects were asked to report their pain ratings on the VAS slider (0 'no pain', 100 'worst imaginable pain'). Here we report the descriptive analysis of VAS for each experiment as well as all the analysis we run. The statistical analysis methods used are the same reported in paragraph 3.5 of the main manuscript and above.

SECTION II – EXPERIMENT 1

EXPERIMENT 1A

(1) Questionnaire

EXPERIMENT 1A – Questionnaire results								
Comparison	***Statement***	***Statistic***	***95% CI***	***p value***	***p value$_{BH}$***	***Effect Size***	***Bayes Factor***	***Power (1-β)***
Congruent vs. Incongruent	*S1*	t = 3.06	0.38, 1.89	0.005**	0.01*	d_z = 0.56	BF_{10} = 8.5	0.84
	S2	V = 149	1, 3	0.005**	0.01*	r_C = 0.74	BF_{10} = 8.49	1
	S5	V = 345.5	3.5, 5.5	0***	0***	r_C = 0.97	BF_{10} = 673474.56	1
	S6	t = 4.35	1.02, 2.84	0***	0***	d_z = 0.79	BF_{10} = 176.19	0.99

Table S1*. *Analysis questionnaire, experiment 1A. *t = t test statistics, V = Wilcoxon sign rank statistic, BH = Bonferroni-Holm correction, d_z = Cohen d_z effect size, r_C = paired rank-biserial correlation effect size, BF_{10} = bayes factor in favour of the alternative hypothesis, BF_{01} = bayes factor in favour of the null hypothesis.*
Importantly the Bonferroni-Holm correction was applied for 4 comparisons.
*Note: * = $p < 0.05$, ** = $p < 0.01$, *** = $p < 0.001$*

(2) VAS

EXPERIMENT 1A – VAS descriptive			
Variable	***Level***	***Mean (± SD)***	***Median (1Q~3Q)***
**Condition**	*Congruent*	26.78 (±17.04)	23.2 (14.17~36.92)
	Incongruent	28.61 (±18.69)	23.3 (17~37.35)
**Gender**	*Female*	28.66 (±18.71)	22.3 (17.4~31.05)
	Male	26.85 (±15.54)	26.3 (16.61~33.79)
**Block**	*Block 1*	28.8 (±17.84)	24.9 (15.93~39.17)
	Block 2	26.59 (±17.9)	22.5 (14.25~36.38)

Table S2*. *VAS descriptive statistics, experiment 1A. *Descriptive statistics of the VAS scores during experiment 1A are shown. SD = standard deviation, 1Q ~ 3Q =first quartile ~ third quartile.*

EXPERIMENT 1A – VAS analysis							
Comparison	***Statistic***	***95% CI***	***p value***	***p value*** $_{BH}$	***Effect Size***	***Bayes Factor***	***Power (1-β)***
Congruent vs. Incongruent	t = -0.82	-6.37, 2.73	0.419	0.978	d_z = -0.15	BF_{01} = 3.78	0.12
Block 1 vs. 2	t = 1	-2.31, 6.73	0.326	0.978	d_z = 0.18	BF_{01} = 3.26	0.16
Female vs. Male	W = 112	-13.5, 10.5	1	1	r_G = 0.01	BF_{01} = 2.81	0.05

Table S3*. *Analysis VAS, experiment 1A. *t = t test statistics, W = Wilcoxon rank sum statistic, BH = Bonferroni-Holm correction, d_z = Cohen d_z effect size, r_G = Glass biserial correlation coefficient effect size, BF_{10} = bayes factor in favour of the alternative hypothesis, BF_{01} = bayes factor in favour of the null hypothesis.*
Importantly the Bonferroni-Holm correction was applied for 3 comparisons.
*Note: * = p < 0.05, ** = p < 0.01, *** = p < 0.001*

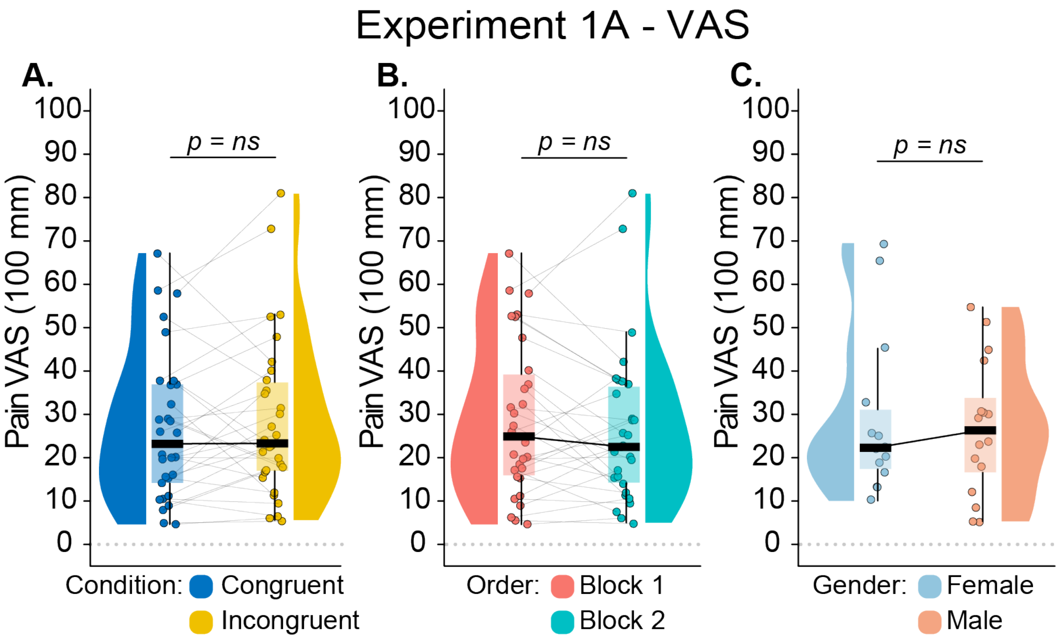

***Figure S1*. *Raincloud plots VAS data, experiment 1A*.** *Paired raincloud plots show individual data points in the Visual Analogue Scale (VAS) evaluation during experiment 1A. **(A.)** The raincloud plot shows the VAS individual data points in the comparison between the two conditions, i.e., Congruent vs. Incongruent. **(B.)** The raincloud plot shows the VAS individual data points in the comparison between the two ordered blocks, i.e., Block 1 vs. Block2. **(C.)** The raincloud plot shows the averaged VAS individual data points in the comparison between the two genders, i.e., Female vs. Male.*
*Note: * = p < 0.05, ** = p < 0.01, *** = p < 0.001*

EXPERIMENT 1B

(1) Proprioceptive drift

EXPERIMENT 1B – Drift analysis						
Comparison	*Statistic*	*95% CI*	*p value*	*Effect Size*	*Bayes Factor*	*Power (1-β)*
Congruent vs. Incongruent	t = 2.72	0.23, 1.59	0.011*	$d_z = 0.5$	$BF_{10} = 4.22$	0.75

Table S4*. *Analysis proprioceptive drift, experiment 1B. *t = t test statistics, d_z = Cohen d_z effect size, BF_{10} = bayes factor in favour of the alternative hypothesis.*
*Note: * = p < 0.05, ** = p < 0.01, *** = p < 0.001*

(2) VAS

EXPERIMENT 1B – VAS descriptive			
Variable	*Level*	*Mean (± SD)*	*Median (1Q~3Q)*
**Condition**	*Congruent*	25.01 (±14.12)	20.28 (14.97~35.75)
	Incongruent	23.62 (±13.15)	20.3 (12.35~33.89)
**Gender**	*Female*	22.76 (±13.75)	18.07 (11.93~34.09)
	Male	27.01 (±13.1)	25.95 (17.52~33.46)
**Block**	*Block 1*	27.7 (±16.5)	27.35 (17.55~36.33)
	Block 2	23.89 (±15.75)	23.65 (12.47~29.12)
	Block 3	24.64 (±16.44)	16.45 (11.97~38.48)
	Block 4	22.02 (±11.96)	20.05 (13.6~30.2)
	Block 5	24.7 (±14.14)	22.6 (12.97~36.8)
	Block 6	22.94 (±13.9)	18.2 (12.05~33.5)

Table S5*. *VAS Descriptive statistics, experiment 1B. *Descriptive statistics of the VAS scores during experiment 1B are shown. SD = standard deviation, 1Q ~ 3Q =first quartile ~ third quartile.*

EXPERIMENT 1B – VAS analysis							
Comparison	***Statistic***	***95% CI***	***p value***	***p value*** $_{BH}$	***Effect Size***	***Bayes Factor***	***Power (1-β)***
Congruent vs. Incongruent	$t = 1.67$	-0.31, 3.1	0.105	0.554	$d_z = 0.31$	$BF_{01} = 1.49$	0.37
Block 1 vs. 2	V = 314	0.15, 6.15	0.037*	1	$r_C = 0.44$	$BF_{10} = 1.5$	0.71
Block 1 vs. 3	$t = 1.53$	-1.04, 7.17	0.137	0.018*	$d_z = 0.28$	$BF_{01} = 1.81$	0.31
Block 1 vs. 4	V = 392	2, 8.1	0.001**	1	$r_C = 0.69$	$BF_{10} = 18.16$	0.99
Block 1 vs. 5	$t = 1.33$	-1.6, 7.61	0.193	0.052	$d_z = 0.24$	$BF_{01} = 2.31$	0.25
Block 1 vs. 6	$t = 3.21$	1.73, 7.81	0.003**	1	$d_z = 0.59$	$BF_{10} = 11.73$	0.87
Block 2 vs. 3	$t = -0.46$	-4.08, 2.59	0.65	1	$d_z = -0.08$	$BF_{01} = 4.67$	0.07
Block 2 vs. 4	$t = 1.15$	-1.46, 5.2	0.261	1	$d_z = 0.21$	$BF_{01} = 2.83$	0.2
Block 2 vs. 5	$t = -0.35$	-5.49, 3.88	0.727	1	$d_z = -0.06$	$BF_{01} = 4.86$	0.06
Block 2 vs. 6	$t = 0.51$	-2.86, 4.77	0.612	1	$d_z = 0.09$	$BF_{01} = 4.56$	0.08
Block 3 vs. 4	$t = 1.53$	-0.88, 6.11	0.137	1	$d_z = 0.28$	$BF_{01} = 1.81$	0.32
Block 3 vs. 5	$t = -0.03$	-4.38, 4.26	0.978	1	$d_z = -0.01$	$BF_{01} = 5.14$	0.05
Block 3 vs. 6	$t = 0.94$	-2, 5.41	0.355	0.554	$d_z = 0.17$	$BF_{01} = 3.44$	0.15
Block 4 vs. 5	$t = -2.19$	-5.18, -0.17	0.037*	1	$d_z = -0.4$	$BF_{10} = 1.52$	0.56
Block 4 vs. 6	V = 163	-3.6, 0.7	0.153	1	$r_C = -0.3$	$BF_{01} = 4.32$	0.37
Block 5 vs. 6	$t = 1.06$	-1.65, 5.18	0.299	1	$d_z = 0.19$	$BF_{01} = 3.09$	0.18
Female vs. Male	W = 124	-6.07, 15.45	0.42	0.554	$r_G = -0.19$	$BF_{01} = 2.19$	0.17

Table S6**. **Analysis VAS, experiment 1B. *t = t test statistics, V = Wilcoxon sign rank statistic, W = Wilcoxon rank sum statistic, BH = Bonferroni-Holm correction,* d_z *= Cohen* d_z *effect size,* r_C *= paired rank-biserial correlation effect size,* r_G *= Glass biserial correlation effect size,* BF_{10} *= bayes factor in favour of the alternative hypothesis,* BF_{01} *= bayes factor in favour of the null hypothesis.*
Importantly the Bonferroni-Holm correction was applied for 17 comparisons.
*Note: * = p < 0.05, ** = p < 0.01, *** = p < 0.001*

There was no difference in pain ratings between conditions ($t_{29} = 1.67$, $p = 0.105$, $p_{BH} = 0.554$, 95% CI = [-0.31, 3.1], $BF_{10} = 1.49$, $d_z = 0.31$), nor between genders ($W = 124$, $p = 0.42$, $p_{BH} = 0.554$, 95% CI = [-6.07, 15.45], $BF_{01} = 2.19$, $r_G = -0.04$). In addition, there was no main difference among blocks except for some comparison. Pain in block 1 was significantly higher as compared to pain in block 2 ($V = 314$, $p = 0.037$, $p_{BH} = 1$, 95% CI = [0.15, 6.15], $BF_{10} = 1.5$, $r_C = 0.44$), as compared to pain in Block 4 ($V = 392$, $p = 0.001$, $p_{BH} = 1$, 95% CI = [2, 8.1], $BF_{10} = 18.16$, $r_C = 0.69$), and as compared to pain in Block 6 ($t_{29} = 3.21$, $p = 0.003$, $p_{BH} = 1$, 95% CI = [1.73, 7.81], $BF_{10} = 11.73$, $d_z = 0.59$). Also, the felt pain in Block 4 was significantly lower as compared to the pain felt in Block 5 ($t_{29} = -2.19$, $p = 0.037$, $p_{BH} = 1$, 95% CI = [-5.18, -0.17], $BF_{10} = 1.52$, $d_z = -0.4$). In addition, there was no difference in the felt pain between females and males ($W = 124$, $p = 0.42$, $p_{BH} = 0.554$, 95% CI = [-6.07, 15.45], $BF_{10} = -0.04$, $r_G = -0.19$).
See Table S2 for the descriptive statistics and Figure S2 to visualize the individual data points.

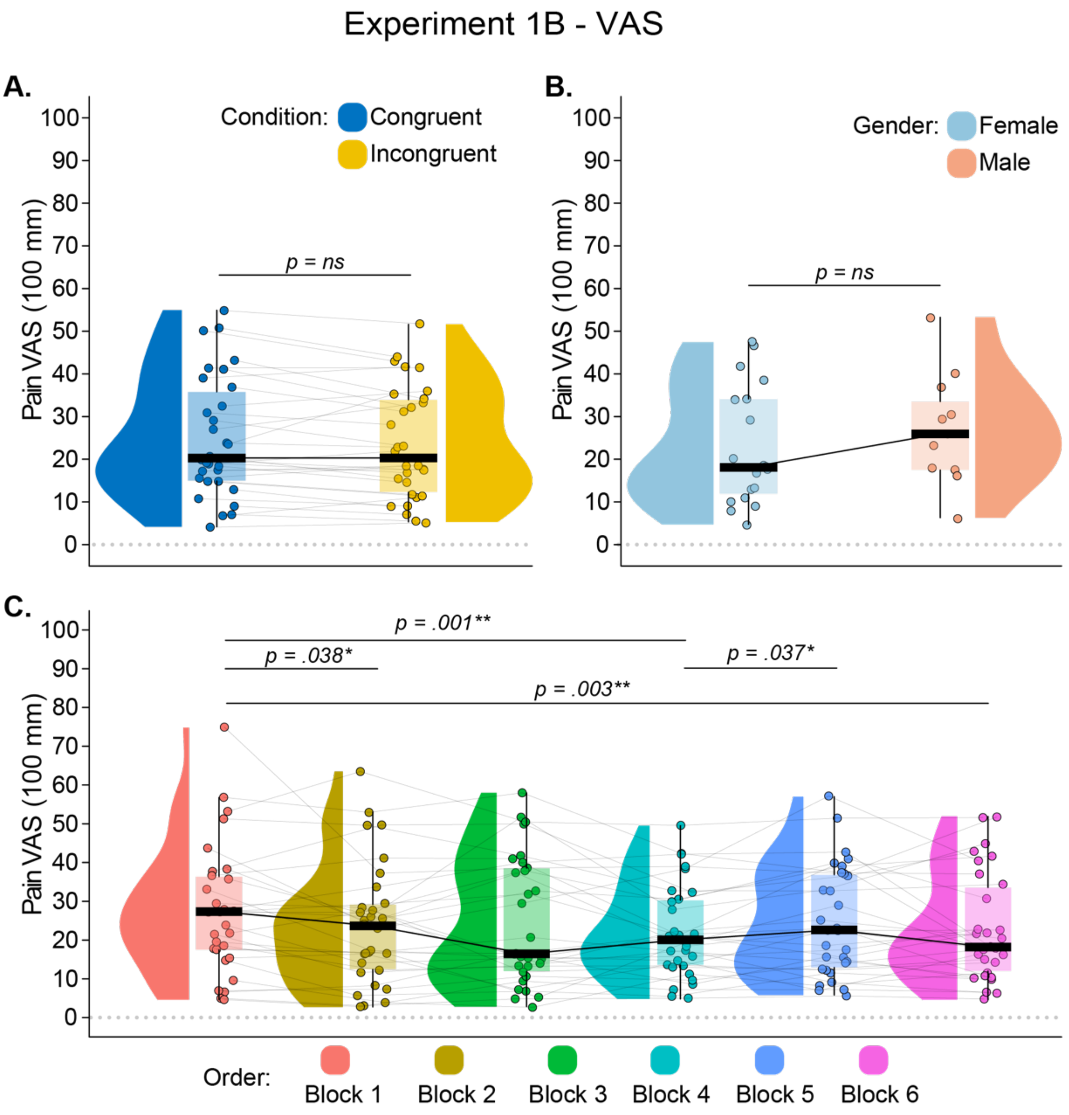

***Figure S2*. *Raincloud plots in the VAS, Experiment 1B*.** *Paired raincloud plots show individual data points in the Visual Analogue Scale (VAS) evaluation during experiment 1B. (**A.**) The raincloud plot shows the VAS individual data points in the comparison between the two conditions, i.e., congruent vs. incongruent. (**B.**) The raincloud plot shows the averaged VAS individual data points in the comparison between the two genders, i.e., Female vs. Male. (**C.**) The raincloud plot shows the VAS individual data points in the comparison between the two ordered blocks, i.e., Block 1 vs. Block 2 vs. Block 3 vs. Block 4 vs. Block 5 vs. Block 6.*

*Note: * = p < 0.05, ** = p < 0.01, *** = p < 0.001, uncorrected*

SECTION III – EXPERIMENT 2

EXPERIMENT 2A

(1) Questionnaire

EXPERIMENT 2A – Questionnaire analysis								
Comparison	***Statement***	***Statistic***	***95% CI***	***p value***	***p value$_{BH}$***	***Effect Size***	***Bayes Factor***	***Power (1-β)***
Congruent vs. Rotated	*S1*	$t = 3.58$	0.59, 2.15	0.001**	0.004**	$d_z = 0.65$	$BF_{10} = 27.35$	0.93
	S2	V = 186	1.5, 3.5	0***	0.001**	$r_C = 0.96$	$BF_{10} = 399.91$	1
	S5	V = 94.5	0, 1.5	0.158	0.158	$r_C = 0.39$	$BF_{01} = 1.58$	0.58
	S6	V = 155.5	0.5, 2	0.013*	0.026	$r_C = 0.64$	$BF_{10} = 2.36$	0.98

Table S7*. *Analysis questionnaire, experiment 2A. *t = t test statistics, V = Wilcoxon sign rank statistic, BH = Bonferroni-Holm correction, d_z = Cohen d_z effect size, r_C = paired rank-biserial correlation effect size, BF_{10} = bayes factor in favour of the alternative hypothesis, BF_{01} = bayes factor in favour of the null hypothesis.*
Importantly the Bonferroni-Holm correction was applied for 4 comparisons.
*Note: * = p < 0.05, ** = p < 0.01, *** = p < 0.001*

(2) VAS

EXPERIMENT 2A – VAS descriptive			
Variable	***Level***	***Mean (± SD)***	***Median (1Q~3Q)***
**Condition**	*Congruent*	20.69 (±8.04)	19.15 (15.43~26.67)
	Rotated	21.84 (±10.09)	19.15 (16.6~27.42)
**Gender**	*Female*	21.3 (±5.99)	22.6 (16.08~25.49)
	Male	21.24 (±7.17)	19.62 (16.14~26.52)
**Block**	*Block 1*	22.12 (±9.5)	20.35 (16.67~26.67)
	Block 2	20.41 (±8.68)	18.65 (14.32~27.42)

Table S8*. *VAS Descriptive statistics, experiment 2A. *Descriptive statistics of the VAS scores during experiment 2A are shown. SD = standard deviation, 1Q ~ 3Q =first quartile ~ third quartile.*

EXPERIMENT 2A – VAS analysis							
Comparison	***Statistic***	***95% CI***	***p value***	***p value*** $_{BH}$	***Effect Size***	***Bayes Factor***	***Power (1-β)***
Congruent vs. Rotated	V = 213	-3.05, 2.9	0.922	1	$r_C = -0.02$	$BF_{01} = 4.6$	0.05
Block 1 vs. 2	V = 222	-2.6, 3.55	0.922	1	$r_C = 0.02$	$BF_{01} = 4$	0.05
Female vs. Male	$t = -0.02$	-4.99, 4.86	0.982	1	$d_s = -0.01$	$BF_{01} = 2.9$	0.05

***Table S9**. **Analysis VAS, experiment 2A.** t = t test statistics, V = Wilcoxon sign rank statistic, BH = Bonferroni-Holm correction,* d_s *= Cohen* d_s *effect size,* r_C *= paired rank-biserial correlation effect size,* BF_{10} *= bayes factor in favour of the alternative hypothesis,* BF_{01} *= bayes factor in favour of the null hypothesis.*
Importantly the Bonferroni-Holm correction was applied for 3 comparisons.
*Note: * = p < 0.05, ** = p < 0.01, *** = p < 0.001*

There was no difference in pain ratings between conditions ($V = 213$, $p = 0.922$, $p_{BH} = 1$, 95% CI = [-3.05, 2.9], $BF_{01} = 4.6$, $r_C = -0.02$), nor between blocks ($V = 222$, $p = 0.931$, $p_{BH} = 0.922$, $p_{BH} = 1$, 95% CI = [-2.6, 3.55], $BF_{01} = 4$, $r_C = 0.02$), nor between genders ($t = -0.02$, $p = 0.982$, $p_{BH} = 0.982$, $p_{BH} = 1$, 95% CI = [-4.99, 4.86], $BF_{01} = 2.9$, $d_s = -0.01$). See Table S3 for the descriptive statistics and Figure S3 to visualize the individual data points.

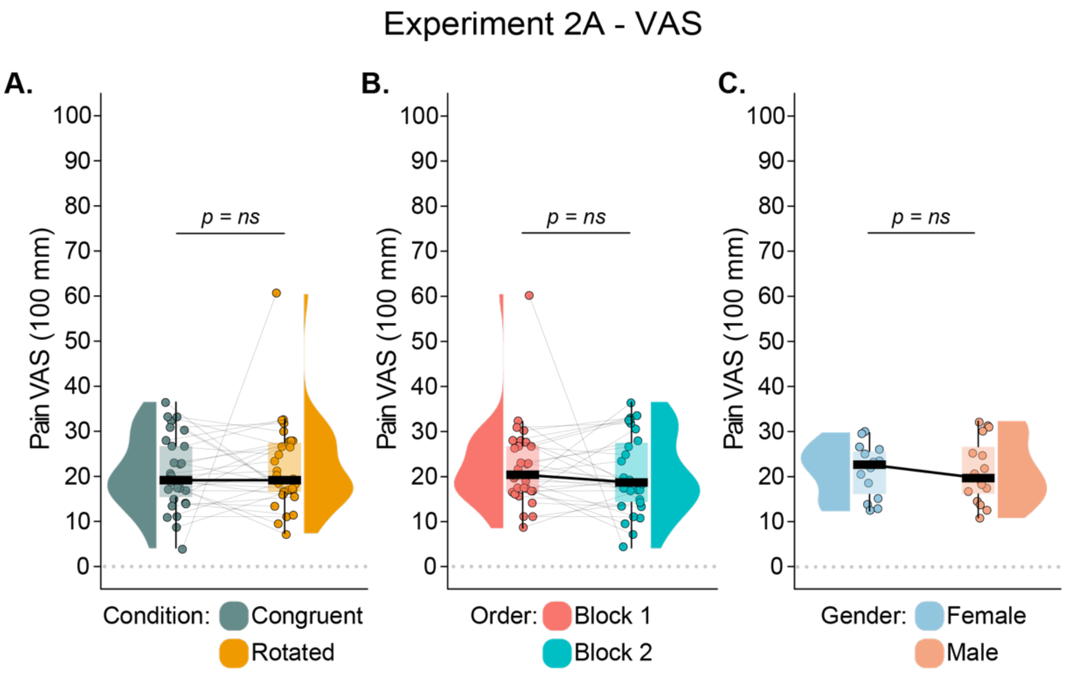

***Figure S3**. **Raincloud plots VAS data, Experiment 2A.** Paired raincloud plots show individual data points in the Visual Analogue Scale (VAS) evaluation during experiment 2A. (**A.**) The raincloud plot shows the VAS individual data points in the comparison between the two conditions, i.e., congruent (= RH at 0°) vs. rotated (= RH at 180°). (**B.**) The raincloud plot shows the VAS individual data points in the comparison between the two ordered blocks, i.e., Block 1 vs. Block2. (**C.**) The raincloud plot shows the averaged VAS individual data points in the comparison between the two genders, i.e., Female vs. Male.*
*Note: * = p < 0.05, ** = p < 0.01, *** = p < 0.001*

EXPERIMENT 2B

(1) Proprioceptive drift

EXPERIMENT 2B – Drift analysis						
Comparison	***Statistic***	***95% CI***	***p value***	***Effect Size***	***Bayes Factor***	***Power (1-β)***
Congruent vs. Rotated	$t = 3.6$	0.71, 2.58	0.001**	$d_z = 0.66$	$BF_{10} = 29.14$	0.94

Table S10**. **Analysis proprioceptive drift, experiment 2B. *t = t test statistics, d_z = Cohen d_z effect size, BF_{10} = bayes factor in favour of the alternative hypothesis, BF_{01} = bayes factor in favour of the null hypothesis.*
*Note: * = $p < 0.05$, ** = $p < 0.01$, *** = $p < 0.001$*

(2) VAS

EXPERIMENT 2B – VAS descriptive			
Variable	***Level***	***Mean (± SD)***	***Median (1Q~3Q)***
**Condition**	*Congruent*	23.25 (±14.75)	18.58 (14.52~28.48)
	Rotated	25.98 (±17.18)	19.85 (13.19~37.69)
**Gender**	*Female*	19.32 (±13.04)	14.97 (12.22~24.1)
	Male	26.89 (±16.27)	20.28 (17.52~32.87)
**Block**	*Block 1*	27.06 (±16.56)	24.4 (14.7~38.03)
	Block 2	26.17 (±18.36)	20.5 (11.15~32.78)
	Block 3	25.77 (±17.48)	19.8 (12.85~32.6)
	Block 4	23.12 (±16.45)	19.15 (12.55~27.45)
	Block 5	23.74 (±17.06)	18.3 (11.75~32.6)
	Block 6	21.85 (±16.57)	16.7 (10.5~30.15)

***Table S11**. **VAS Descriptive statistics, experiment 2B.** Descriptive statistics of the VAS values during experiment 2B are shown. SD = standard deviation, 1Q ~ 3Q =first quartile ~ third quartile.*

**EXPERIMENT 2B – VAS analysis**							
Comparison	***Statistic***	***95% CI***	***p value***	***p value*** $_{BH}$	***Effect Size***	***Bayes Factor***	***Power (1-β)***
Congruent vs. Rotated	V = 147.5	-3.2, 0.13	0.08	0.965	r_C = -0.37	BF_{01} = 0.96	0.52
Block 1 vs. 2	t = 0.42	-3.37, 5.14	0.674	1	d_z = 0.08	BF_{01} = 4.73	0.07
Block 1 vs. 3	t = 0.85	-1.8, 4.38	0.401	1	d_z = 0.16	BF_{01} = 3.69	0.13
Block 1 vs. 4	t = 2.32	0.46, 7.42	0.028*	0.472	d_z = 0.42	BF_{10} = 1.92	0.61
Block 1 vs. 5	t = 1.68	-0.73, 7.36	0.104	1	d_z = 0.31	BF_{01} = 1.48	0.37
Block 1 vs. 6	V = 328	-0.05, 7.5	0.049*	0.742	r_C = 0.41	BF_{01} = 0.86	0.63
Block 2 vs. 3	t = 0.17	-4.4, 5.2	0.865	1	d_z = 0.03	BF_{01} = 5.07	0.05
Block 2 vs. 4	V = 269	-1.4, 5.8	0.265	1	r_C = 0.24	BF_{01} = 1.77	0.25
Block 2 vs. 5	t = 0.95	-2.81, 7.67	0.35	1	d_z = 0.17	BF_{01} = 3.41	0.15
Block 2 vs. 6	t = 1.91	-0.3, 8.95	0.066	0.856	d_z = 0.35	BF_{01} = 1.04	0.46
Block 3 vs. 4	V = 324	-0.3, 4.9	0.06	0.838	r_C = 0.39	BF_{01} = 2.17	0.59
Block 3 vs. 5	t = 1.51	-0.72, 4.78	0.141	1	d_z = 0.28	BF_{01} = 1.85	0.31
Block 3 vs. 6	V = 311	0.05, 6.65	0.043*	0.691	r_C = 0.43	BF_{10} = 1.09	0.68
Block 4 vs. 5	V = 216	-2.25, 1.9	0.734	1	r_C = -0.07	BF_{01} = 4.82	0.07
Block 4 vs. 6	V = 228.5	-2.8, 3.2	0.812	1	r_C = 0.05	BF_{01} = 4.25	0.06
Block 5 vs. 6	V = 259	-2.5, 3.75	0.586	1	r_C = 0.11	BF_{01} = 3.21	0.09
Female vs. Male	W = 127	-3.82, 17.33	0.15	1	r_G = 0.34	BF_{01} = 1.57	0.47

***Table S12**. **Analysis VAS, experiment 2B.** t = t test statistics, V = Wilcoxon sign rank statistic, W = Wilcoxon rank sum statistic, BH = Bonferroni-Holm correction,* d_z *= Cohen* d_z *effect size,* r_C *= paired rank-biserial correlation effect size,* r_G *= Glass biserial correlation effect size,* BF_{10} *= bayes factor in favour of the alternative hypothesis,* BF_{01} *= bayes factor in favour of the null hypothesis.*
Importantly the Bonferroni-Holm correction was applied for 17 comparisons.
*Note: * = p < 0.05, ** = p < 0.01, *** = p < 0.001*

There was no difference in pain ratings between conditions (V = 147.5, p = 0.08, p_{BH} = 0.965, 95% CI = [-3.2, 0.13], BF_{01} = 0.96, r_C = -0.37), nor between genders (W = 127, p = 0.15, p_{BH} = 1, 95% CI = [-3.82, 17.33], BF_{01} = 1.57, r_G = 0.34). In addition, there was no main difference among blocks except for some comparisons. Pain in block 1 was significantly higher as compared to pain in block 4 (t = 2.32, p = 0.028, p_{BH} = 0.472, 95% CI = [0.46, 7.42], BF_{10} = 1.92, d_z = 0.42). The felt pain in Block 3 was significantly higher as compared to the pain felt in Block 6 (V = 311, p = 0.044, p_{BH} = 0.691, 95% CI = [0.05, 6.65], BF_{10} = 1.09, r_C = 0.43).
See Table S4 for the descriptive statistics and Figure S4 to visualize the individual data points.

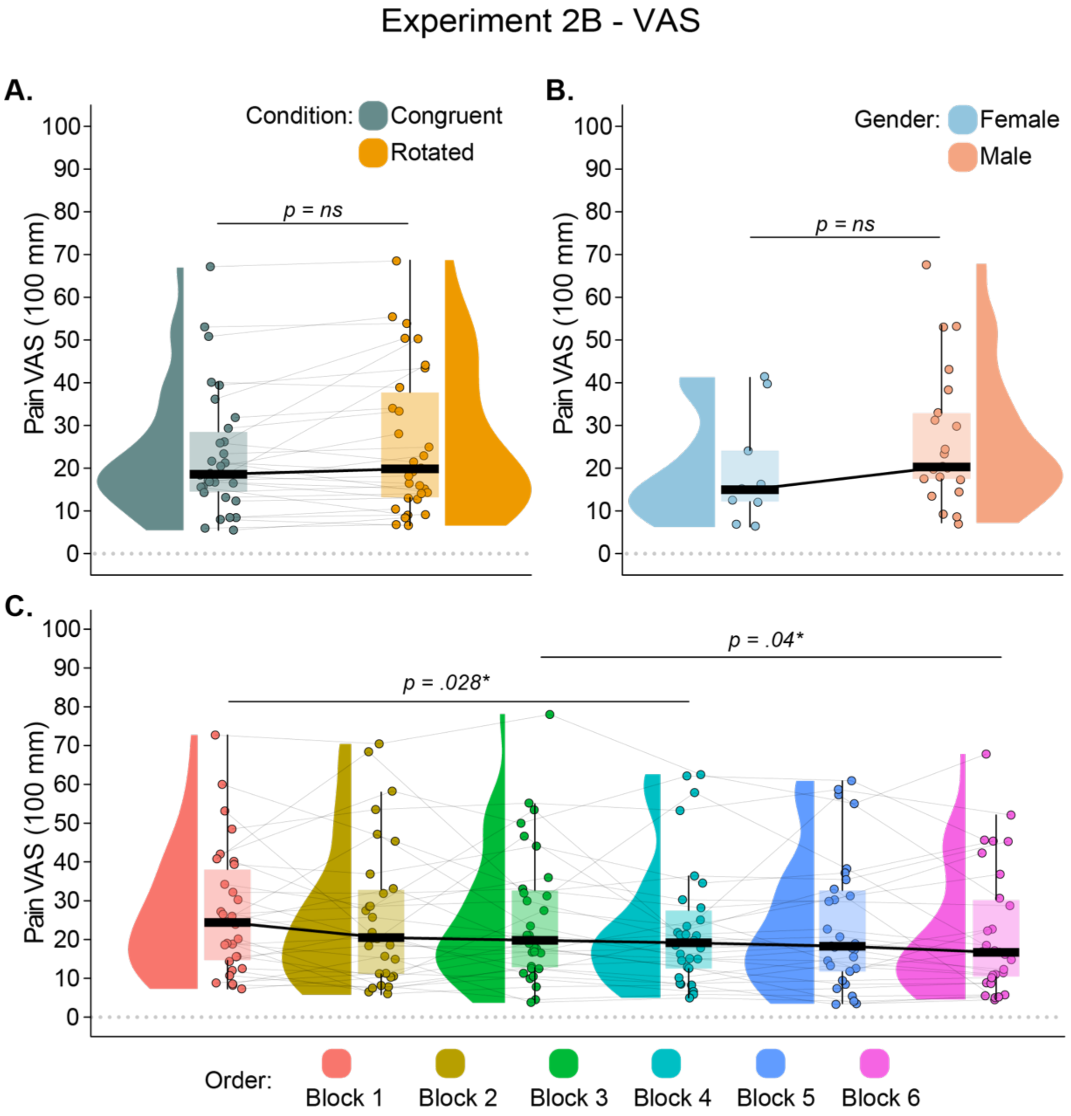

Figure S4*. *Raincloud plots for VAS data, Experiment 2B. *Paired raincloud plots show individual data points in the Visual Analogue Scale (VAS) evaluation during experiment 2B. (**A.**) The raincloud plot shows the average VAS individual data points in the comparison between the two conditions, i.e., congruent (= RH at 0°) vs. rotated (= RH at 180°). (**B.**) The raincloud plot shows the averaged VAS individual data points in the comparison between the two genders, i.e., Female vs. Male. (**C.**) The raincloud plot shows the VAS individual data points in the comparison between the two ordered blocks, i.e., Block 1 vs. Block 2 vs. Block 3 vs. Block 4 vs. Block 5 vs. Block 6.*

*Note: * = p < 0.05, ** = p < 0.01, *** = p < 0.001, uncorrected*

SECTION IV – EXPERIMENT 3

EXPERIMENT 3A

(1) Questionnaire

EXPERIMENT 3A – Questionnaire analysis

Comparison	*Statement*	*Statistic*	*95% CI*	*p value*	*p value* $_{BH}$	*Effect Size*	*Bayes Factor*	*Power (1-β)*
Congruent vs. Incongruent	S1	V = 149	1, 3	0.001**	**0.006	r_C = 0.95	BF_{10} = 82.11	1
	S2	V = 201	1, 2.5	0.002**	*0.022	r_C = 0.74	BF_{10}= 21.49	1
	S5	V = 325	3.5, 4.5	0***	***0	r_C = *1*	BF_{10} = 29812186.94	1
	S6	*t* = 4.6	1.07, 2.79	0***	**0.001	d_z = *0.84*	BF_{10} = 326.44	0.99
Congruent vs. Hand	S1	V = 86	-1.5, 1	0.982	1	r_C = 0.01	BF_{01} = 5.14	0.05
	S2	V = 103.5	-1.5, 1	0.671	1	r_C = -0.1	BF_{01} = 4.87	0.08
Congruent vs. Light	S1	*t* = 1.4	-0.24, 1.31	0.171	0.785	d_z = 0.26	BF_{01} = 2.12	0.27
	S2	V = 104.5	-0.5, 3	0.182	0.785	r_C = 0.37	BF_{01} = 2.12	0.53
Light vs. Incongruent	S1	V = 127	0, 2.5	0.066	0.531	r_C = 0.49	BF_{01} = 0.9	0.8
	S2	*t* = 1.69	-0.12, 1.25	0.101	0.606	d_z = 0.31	BF_{01} = 1.44	0.37
Hand vs. Incongruent	S1	V = 192	1, 2.5	0.001**	*0.01	r_C = 0.83	BF_{10} = 37.72	1
	S2	V = 171.5	1, 3	0.002**	*0.019	r_C = 0.81	BF_{10} = 32.49	1
Hand vs. Light	S1	V = 95	-0.5, 2.5	0.157	0.785	r_C = 0.4	BF_{01} = 1.7	0.6
	S2	V = 164.5	0, 2.5	0.084	0.589	r_C = 0.42	BF_{01} = 0.96	0.67

Table S13*. *Analysis questionnaire, experiment 3A. *t = t test statistics, V = Wilcoxon sign rank statistic, BH = Bonferroni-Holm correction,* d_z *= Cohen* d_z *effect size,* r_C *= paired rank-biserial correlation effect size,* BF_{10} *= bayes factor in favour of the alternative hypothesis,* BF_{01} *= bayes factor in favour of the null hypothesis.*
Importantly the Bonferroni-Holm correction was applied for 14 comparisons.
*Note: * = p < 0.05, ** = p < 0.01, *** = p < 0.001*

(2) VAS

EXPERIMENT 3A – VAS descriptive

Variable	*Level*	*Mean (± SD)*	*Median (1Q~3Q)*
**Condition**	*Congruent*	20.45 (±8.69)	19.9 (15.03~24.22)
	Incongruent	19.53 (±9.87)	18.45 (15.03~21.6)
**Gender**	*Female*	20.2 (±9.07)	17 (15.96~22.49)
	Male	19.74 (±8.76)	20 (15.58~24.06)
**Block**	*Block 1*	19.87 (±9.05)	19.7 (16.45~22.2)
	Block 2	20.11 (±9.57)	18.2 (14.35~23.42)

Table S14*. *VAS Descriptive statistics, experiment 3A. *Descriptive statistics of the VAS scores during experiment 3A are shown. SD = standard deviation, 1Q ~ 3Q =first quartile ~ third quartile.*

Table S15.

EXPERIMENT 3A – VAS analysis							
Comparison	***Statistic***	***95% CI***	***p value***	***p value*** **BH**	***Effect Size***	***Bayes Factor***	***Power (1-β)***
Congruent vs. Incongruent	$t = 0.82$	-1.37, 3.22	0.417	1	$d_z = 0.15$	$BF_{01} = 3.77$	0.13
Block 1 vs. 2	$t = -0.21$	-2.55, 2.08	0.836	1	$d_z = -0.04$	$BF_{01} = 5.04$	0.05
Female vs. Male	W = 119	-6.3, 6.15	0.79	1	$r_G = -0.06$	$BF_{01} = 2.88$	0.06

Analysis VAS, experiment 3A. *t = t test statistics, W = Wilcoxon rank sum statistic, BH = Bonferroni-Holm correction,* d_z *= Cohen* d_z *effect size,* r_G *= Glass biserial correlation effect size,* BF_{10} *= bayes factor in favour of the alternative hypothesis,* BF_{01} *= bayes factor in favour of the null hypothesis.*
Importantly the Bonferroni-Holm correction was applied for 3 comparisons.
*Note: * = p < 0.05, ** = p < 0.01, *** = p < 0.001*

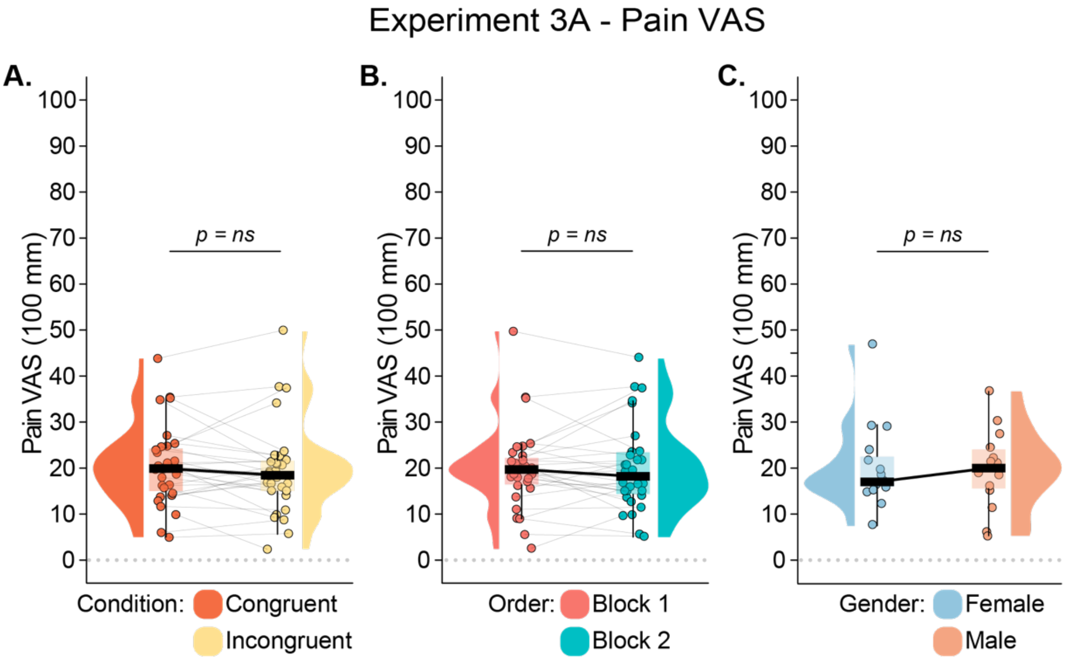

Figure S5**.** ***Raincloud plots VAS data, Experiment 3A****. Paired raincloud plots show individual data points in the Visual Analogue Scale (VAS) evaluation during experiment 3A.* ***(A.)*** *The raincloud plot shows the VAS individual data points in the comparison between the two painful conditions, i.e., congruent vs. incongruent.* ***(B.)*** *The raincloud plot shows the VAS individual data points in the comparison between the two ordered painful blocks, i.e., Block 1 vs. Block2.* ***(C.)*** *The raincloud plot shows the averaged VAS individual data points in the comparison between the two genders, i.e., Female vs. Male.*
*Note: * = p < 0.05, ** = p < 0.01, *** = p < 0.001*

There was no difference in pain ratings between conditions ($t_{29} = 0.82$, $p = 0.417$, $p_{BH} = 1$, 95% CI = [-1.37, 3.22], $BF_{01} = 0.27$, $d_z = 0.10$), nor between the first painful block and the second painful block ($t_{29} = -0.21$, $p = 0.836$, $p_{BH} = 1$, 95% CI = [-2.55, 2.08], $BF_{01} = 0.12$, $d_z = -0.04$), nor between genders ($W = 119$, $p = 0.79$, $p_{BH} = 1$, 95% CI = [-6.3,6.16], $BF_{01} = 0.35$, $r_G = -0.06$).

(3) EMG Analysis

EXPERIMENT 3A – EMG descriptive			
Variable	***Condition***	***Mean (± SD)***	***Median (1Q~3Q)***
Estensor - 120 sec block	*Congruent*	0.0601 (±0.0037)	0.0606 (0.0561~0.0629)
	Incongruent	0.0599 (±0.0038)	0.0605 (0.0557~0.0634)
	Hand	0.0598 (±0.004)	0.0602 (0.0557~0.0637)
	Light	0.06 (±0.0036)	0.061 (0.0561~0.0627)
Estensor, input for 200ms	*Congruent*	0.0601 (±0.0038)	0.0605 (0.0561~0.0629)
	Incongruent	0.06 (±0.0038)	0.0604 (0.0557~0.0634)
Left bicep - 120 sec block	*Congruent*	0.0617 (±0.0039)	0.0623 (0.058~0.065)
	Incongruent	0.0616 (±0.004)	0.0622 (0.0577~0.0651)
	Hand	0.0614 (±0.0042)	0.0622 (0.0576~0.0653)
	Light	0.0616 (±0.0037)	0.0622 (0.0579~0.0642)
Left bicep, input for 200ms	*Congruent*	0.0617 (±0.004)	0.0623 (0.058~0.0651)
	Incongruent	0.0616 (±0.004)	0.062 (0.0577~0.0651)

***Table S16**. **EMG Descriptive statistics, experiment 3A.** Descriptive statistics of the EMG values during experiment 3A are shown. SD = standard deviation, 1Q ~ 3Q =first quartile ~ third quartile.*

EXPERIMENT 3A – EMG analysis							
RMS	***Comparison***	***Statistic***	***95% CI***	***p value***	***p value BH***	***Effect Size***	***Bayes Factor***
Estensor - 120 sec block	*Congruent vs. Incongruent*	V = 267	0, 0	0.284	1	$r_C = 0.23$	$BF_{01} = 3.17$
	Congruent vs. Hand	*t* = 1.79	0, 0	0.085	1	$d_z = 0.33$	$BF_{01} = 1.24$
	Congruent vs. Light	*t* = 0.52	0, 0	0.604	1	$d_z = 0.1$	$BF_{01} = 4.46$
	Light vs. Incongruent	*t* = 0.36	0, 0	0.72	1	$d_z = 0.07$	$BF_{01} = 4.77$
	Hand vs. Incongruent	*t* = -0.92	0, 0	0.367	1	$d_z = -0.17$	$BF_{01} = 3.45$
	Hand vs. Light	*t* = -1.15	0, 0	0.259	1	$d_z = -0.21$	$BF_{01} = 2.78$
Estensor, input for 200ms	*Congruent vs. Incongruent*	V = 236	0, 0	0.689	1	$r_C = 0.09$	$BF_{01} = 4.57$
Left bicep - 120 sec block	*Congruent vs. Incongruent*	V = 279	0, 0	0.184	1	$r_C = 0.28$	$BF_{01} = 2.73$
	Congruent vs. Hand	*t* = 1.98	0, 0	0.058	0.809	$d_z = 0.37$	$BF_{01} = 0.93$
	Congruent vs. Light	*t* = 0.65	0, 0	0.522	1	$d_z = 0.12$	$BF_{01} = 4.17$
	Light vs. Incongruent	*t* = 0.33	0, 0	0.745	1	$d_z = 0.06$	$BF_{01} = 4.82$
	Hand vs. Incongruent	*t* = -0.94	0, 0	0.355	1	$d_z = -0.17$	$BF_{01} = 3.38$
	Hand vs. Light	*t* = -1.04	0, 0	0.308	1	$d_z = -0.19$	$BF_{01} = 3.1$
Left bicep, input for 200ms	*Congruent vs. Incongruent*	V = 276	0, 0	0.206	1	$r_C = 0.27$	$BF_{01} = 2.55$

***Table S17**. **Analysis EMG, experiment 3A.** t = t test statistics, V = Wilcoxon sign rank statistic, BH = Bonferroni-Holm correction, d_z = Cohen d_z effect size, r_C = paired rank-biserial correlation effect size, BF_{10} = bayes factor in favour of the alternative hypothesis, BF_{01} = bayes factor in favour of the null hypothesis.*
Importantly the Bonferroni-Holm correction was applied for 14 comparisons.
*Note: * = p < 0.05, ** = p < 0.01, *** = p < 0.001*

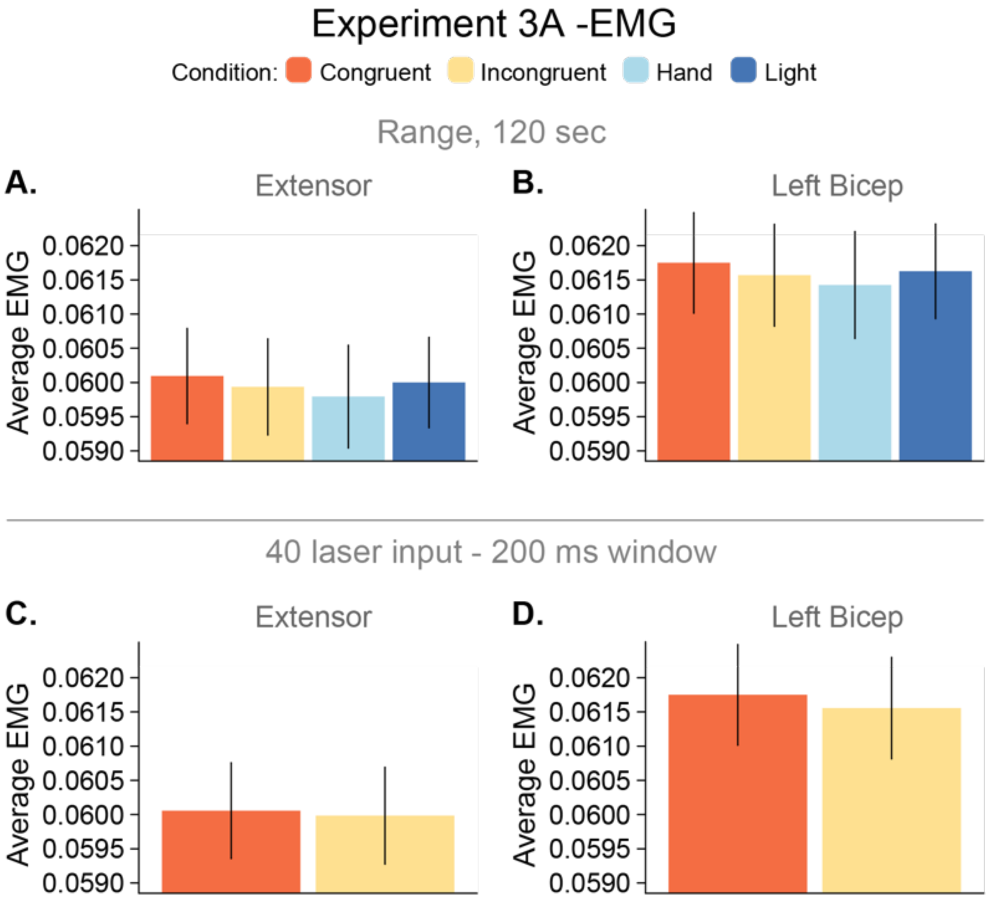

***Figure S6**. **Bar plots EMG data, experiment 3A**. The sample size is 29 since data from one subject were not recorded due to a device failure. In the figure is shown the root mean squared of the EMG activity. (**A.**) The Left Extensor muscular averaged activity across the entire block (i.e., 120 sec) is shown for each condition. (**B**.) The Left Bicep muscular averaged activity across the entire block (i.e., 120 sec) is shown for each condition. (**C.**) The Left Extensor muscular averaged activity after each painful stimulation in the painful blocks, that is the average activity of 40 laser input, is shown. (**D.**) The Left Bicep muscular averaged activity after each painful stimulation in the painful blocks, that is the average activity of 40 laser input, is shown.*

Note: Error bars show standard error and 'rms' stands for 'root mean square'.

*Note: * = $p < 0.05$, ** = $p < 0.01$, *** = $p < 0.001$, uncorrected*

EXPERIMENT 3B

(1) Proprioceptive Drift

EXPERIMENT 3B – Drift analysis							
Comparison	***Statistic***	***95% CI***	***p value***	***p value*** $_{BH}$	***Effect Size***	***Bayes Factor***	***Power (1-β)***
Congruent vs. Incongruent	$t = 3.06$	0.41, 2.04	0.005**	0.028*	$d_z = 0.56$	$BF_{10} = 8.59$	0.84
Congruent vs. Hand	$t = 2.6$	0.21, 1.73	0.015*	0.073*	$d_z = 0.47$	$BF_{10} = 3.27$	0.71
Congruent vs. Light	$t = 2.58$	0.23, 2.02	0.015*	0.073*	$d_z = 0.47$	$BF_{10} = 3.14$	0.7
Light vs. Incongruent	$t = 0.29$	-0.58, 0.77	0.777	1	$d_z = 0.05$	$BF_{01} = 4.95$	0.06
Hand vs. Incongruent	$t = 0.69$	-0.5, 1.01	0.497	1	$d_z = 0.13$	$BF_{01} = 4.14$	0.1
Hand vs. Light	$t = 0.37$	-0.72, 1.04	0.711	1	$d_s = 0.07$	$BF_{01} = 4.82$	0.07

***Table S18**. **Analysis proprioceptive drift, experiment 3B.** t = t test statistics, BH = Bonferroni-Holm correction, d_s = Cohen d_s effect size, BF_{10} = bayes factor in favour of the alternative hypothesis, BF_{01} = bayes factor in favour of the null hypothesis. Importantly the Bonferroni-Holm correction was applied for 6 comparisons.*

*Note: * = $p < 0.05$, ** = $p < 0.01$, *** = $p < 0.001$*

(2) VAS

EXPERIMENT 3B – VAS descriptive			
Variable	***Level***	***Mean (± SD)***	***Median (1Q~3Q)***
**Condition**	*Congruent*	18.84 (±9.43)	16.62 (12.21~23.87)
	Incongruent	18.52 (±8.02)	17.72 (12.98~22.4)
**Gender**	*Female*	8.5 (±3.03)	8.84 (5.84~10.01)
	Male	10.63 (±4.97)	8.82 (7.53~12.72)
**Block**	*Block 1*	16.83 (±6.62)	19.3 (11~21.15)
	Block 2	19.59 (±9.85)	17.15 (13.6~22.82)
	Block 3	17.61 (±6.96)	17 (11.8~23.33)
	Block 4	15.99 (±8.71)	15 (11.5~18.3)
	Block 5	22.81 (±11.05)	21 (15.4~26.9)
	Block 6	20.12 (±9.32)	17.45 (12.43~29.15)

***Table S19**. **VAS Descriptive statistics, experiment 3B.** Descriptive statistics of the VAS values during experiment 3B are shown. SD = standard deviation, 1Q ~ 3Q =first quartile ~ third quartile.*

Comparison	Statistic	95% CI	p value	p value BH	Effect Size	Bayes Factor	Power (1-β)
EXPERIMENT 3B – VAS analysis							
Congruent vs. Incongruent	V = 284.5	-0.12, 0.28	0.285	1	r_C = 0.22	BF_{01} = 4.99	0.22
Block 1 vs. 2	V = 210	-0.27, 0.19	0.644	1	r_C = -0.1	BF_{01} = 3.74	0.08
Block 1 vs. 3	*t* = -2.87	-0.66, -0.11	0.008*	0.106	d_z = -0.52	BF_{10} = 5.71	0.79
Block 1 vs. 4	V = 144	-0.75, 0.03	0.069	0.756	r_C = -0.38	BF_{01} = 1.04	0.56
Block 1 vs. 5	*t* = 0.22	-0.25, 0.31	0.825	1	d_z = 0.04	BF_{01} = 5.03	0.06
Block 1 vs. 6	V = 244	-0.36, 0.3	0.813	1	r_C = 0.05	BF_{01} = 4.24	0.06
Block 2 vs. 3	V = 118	-0.45, -0.03	0.019*	0.241	r_C = -0.49	BF_{10} = 0.69	0.81
Block 2 vs. 4	V = 72	-0.49, -0.12	0.001	0.016*	r_C = -0.69	BF_{10} = 0.44	0.99
Block 2 vs. 5	V = 298	-0.08, 0.33	0.178	1	r_C = 0.28	BF_{01} = 2.69	0.33
Block 2 vs. 6	V = 248	-0.28, 0.24	0.75	1	r_C = 0.07	BF_{01} = 5.12	0.06
Block 3 vs. 4	*t* = -0.37	-0.46, 0.32	0.711	1	d_z = -0.07	BF_{01} = 4.82	0.07
Block 3 vs. 5	*t* = 3.65	0.18, 0.65	0.001**	0.016*	d_z = 0.67	BF_{10} = 32.55	0.94
Block 3 vs. 6	*t* = 1.48	-0.1, 0.62	0.151	1	d_z = 0.27	BF_{01} = 1.94	0.3
Block 4 vs. 5	V = 370	0.14, 0.72	0.005**	0.07	r_C = 0.59	BF_{10} = 3.29	0.95
Block 4 vs. 6	V = 313.5	0.02, 0.53	0.038*	0.455	r_C = 0.44	BF_{10} = 1.6	0.7
Block 5 vs. 6	V = 163	-0.32, 0.1	0.239	1	r_C = -0.25	BF_{01} = 2.68	0.27
Female vs. Male	W = 135	-0.1, 0.42	0.355	1	r_G = -0.21	BF_{01} = 1.34	0.191

Table S20**. **Analysis VAS, experiment 3B. *t = t test statistics, V = Wilcoxon sign rank statistic, W = Wilcoxon rank sum statistic, BH = Bonferroni-Holm correction, d_z = Cohen d_z effect size, r_C = paired rank-biserial correlation effect size, r_G = Glass biserial correlation effect size, BF_{10} = bayes factor in favour of the alternative hypothesis, BF_{01} = bayes factor in favour of the null hypothesis.*
Importantly the Bonferroni-Holm correction was applied for 17 comparisons.
*Note: * = $p < 0.05$, ** = $p < 0.01$, *** = $p < 0.001$*

There was no difference in pain ratings between conditions (*V* = 284.5, *p* = 0.285, p_{BH} = 1, 95% CI = [-1.2, 2.8], BF_{01} = 4.99, r_C = 0.22), nor between genders (*W* = 135, *p* = 0.355, p_{BH} = 1, 95% CI = [-0.1, 0.42], BF_{01} = 1.34, r_g = -0.21). The pain rating was significantly higher in Block 3 as compared to Block 1 (t_{29} = -2.87, *p* = 0.008, p_{BH} = 0.106, 95% CI = [-6.638, -1.115], BF_{10} = 5.71, d_z = -0.52). The pain rating was significantly higher in Block 2 as compared to Block 3 (*V* = 118, *p* = 0.019, p_{BH} = 0.241, 95% CI = [-4.5, -0.03], BF_{10} = 0.69, r_C = -0.49) and as compared to Block 4 (*V* = 72, *p* = 0.001, p_{BH} = 0.016, 95% CI = [-4.9, -1.2], BF_{10} = 0.44, r_C = -0.69). The pain rating in Block 5 was significantly than in Block 3 (t_{29} = 3.65, *p* = 0.001, p_{BH} = 0.016, 95% CI = [1.84, 6.65], BF_{10} = 32.55, d_z = 0.67) and as compared to Block 4 (*V* = 370, *p* = 0.005, p_{BH} = 0.07, 95% CI = [1.4, 7.2], BF_{10} = 3.29, r_C = 0.59). Finally, the pain rating was higher in Block 6 as compared to Block 4 (*V* = 313.5, *p* = 0.038, p_{BH} = 0.455, 95% CI = [0.2, 5.3], BF_{10} = 1.6, r_C = 0.44).

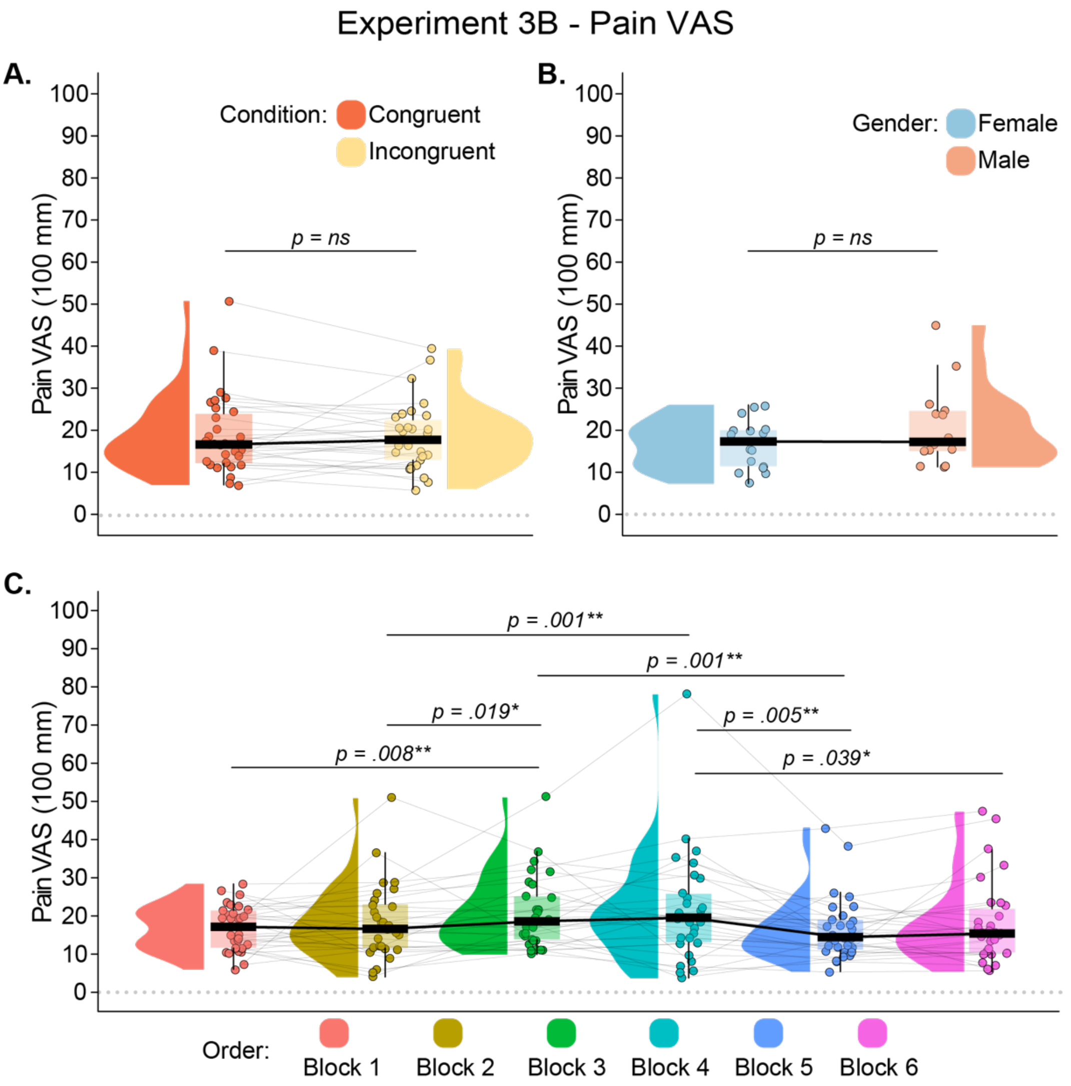

***Figure S7*. Raincloud plots for VAS data, Experiment 3B**. *Paired raincloud plots show individual data points in the Visual Analogue Scale (VAS) evaluation during the painful blocks of experiment 3B. (**A.**) The raincloud plot shows the VAS individual data points in the comparison between the two conditions, i.e., congruent vs. incongruent. (**B.**) The raincloud plot shows the averaged VAS individual data points in the comparison between the two genders, i.e., Female vs. Male. (**C.**) The raincloud plot shows the VAS individual data points in the comparison between the two ordered blocks, i.e., Block 1 vs. Block 2 vs. Block 3 vs. Block 4 vs. Block 5 vs. Block 6.*

*Note: * = p < 0.05, ** = p < 0.01, *** = p < 0.001, uncorrected*

(3) EMG Analysis

EXPERIMENT 3B – EMG descriptive			
Variable	***Condition***	***Mean (± SD)***	***Median (1Q~3Q)***
Estensor - 120 sec block	*Congruent*	0.063 (±0.0052)	0.063 (0.0577~0.0671)
	Incongruent	0.0628 (±0.0055)	0.0637 (0.0578~0.0675)
	Hand	0.0634 (±0.0053)	0.064 (0.0588~0.0676)
	Light	0.063 (±0.0053)	0.0635 (0.0582~0.0678)
Estensor, input for 200ms	*Congruent*	0.063 (±0.0052)	0.063 (0.0577~0.0671)
	Incongruent	0.0628 (±0.0056)	0.0636 (0.0579~0.0674)
Left bicep - 120 sec block	*Congruent*	0.0635 (±0.0051)	0.064 (0.0586~0.0674)
	Incongruent	0.0633 (±0.0055)	0.0645 (0.0588~0.0676)
	Hand	0.064 (±0.0052)	0.0648 (0.0602~0.0682)
	Light	0.0635 (±0.0052)	0.0645 (0.0591~0.0674)
Left bicep, input for 200ms	*Congruent*	0.0635 (±0.0051)	0.064 (0.0586~0.0673)
	Incongruent	0.0633 (±0.0055)	0.0646 (0.0589~0.0675)

***Table S21*. EMG Descriptive statistics, experiment 3B.** *Descriptive statistics of the EMG values during experiment 3B are shown. SD = standard deviation, 1Q ~ 3Q =first quartile ~ third quartile.*

EXPERIMENT 3B – EMG analysis							
RMS	***Comparison***	***Statistic***	***95% CI***	***p value***	***p value $_{BH}$***	***Effect Size***	***Bayes Factor***
Estensor - 120 sec block	*Congruent vs. Incongruent*	V = 257	0, 0	0.393	1	r_C = 0.18	BF_{01} = 3.11
	Congruent vs. Hand	*t* = -2.36	0, 0	0.026*	0.307	d_z = -0.44	BF_{10} = 2.08
	Congruent vs. Light	*t* = -0.13	0, 0	0.899	1	d_z = -0.02	BF_{01} = 5.03
	Light vs. Incongruent	*t* = 1.27	0, 0	0.215	1	d_z = 0.24	BF_{01} = 2.46
	Hand vs. Incongruent	*t* = 2.92	0, 0	0.007**	0.094	d_z = 0.54	BF_{10} = 6.32
	Hand vs. Light	*t* = 2.31	0, 0	0.029*	0.307	d_z = 0.43	BF_{10} = 1.9
Estensor, input for 200ms	*Congruent vs. Incongruent*	V = 258	0, 0	0.381	1	r_C = 0.19	BF_{01} = 3.02
Left bicep - 120 sec block	*Congruent vs. Incongruent*	V = 255	0, 0	0.417	1	r_C = 0.17	BF_{01} = 3.25
	Congruent vs. Hand	*t* = -2.34	0, 0	0.026*	0.307	d_z = -0.44	BF_{10} = 2.02
	Congruent vs. Light	*t* = -0.29	0, 0	0.775	1	d_z = -0.05	BF_{01} = 4.88
	Light vs. Incongruent	*t* = 1.34	0, 0	0.19	1	d_z = 0.25	BF_{01} = 2.25
	Hand vs. Incongruent	*t* = 2.93	0, 0	0.007**	0.094	d_z = 0.54	BF_{10} = 6.4
	Hand vs. Light	*t* = 2.19	0, 0	0.037*	0.33	d_z = 0.41	BF_{10} = 1.55
Left bicep, input for 200ms	*Congruent vs. Incongruent*	V = 254	0, 0	0.43	1	r_C = 0.17	BF_{01} = 3.17

***Table S22*. Analysis EMG, experiment 3B.** *t = t test statistics, V = Wilcoxon sign rank statistic, d_z = Cohen d_z effect size, r_C = paired rank-biserial correlation effect size, BF_{10} = bayes factor in favour of the alternative hypothesis, BF_{01} = bayes factor in favour of the null hypothesis.*
Importantly the Bonferroni-Holm correction was applied for 14 comparisons.
*Note: * = p < 0.05, ** = p < 0.01, *** = p < 0.001*

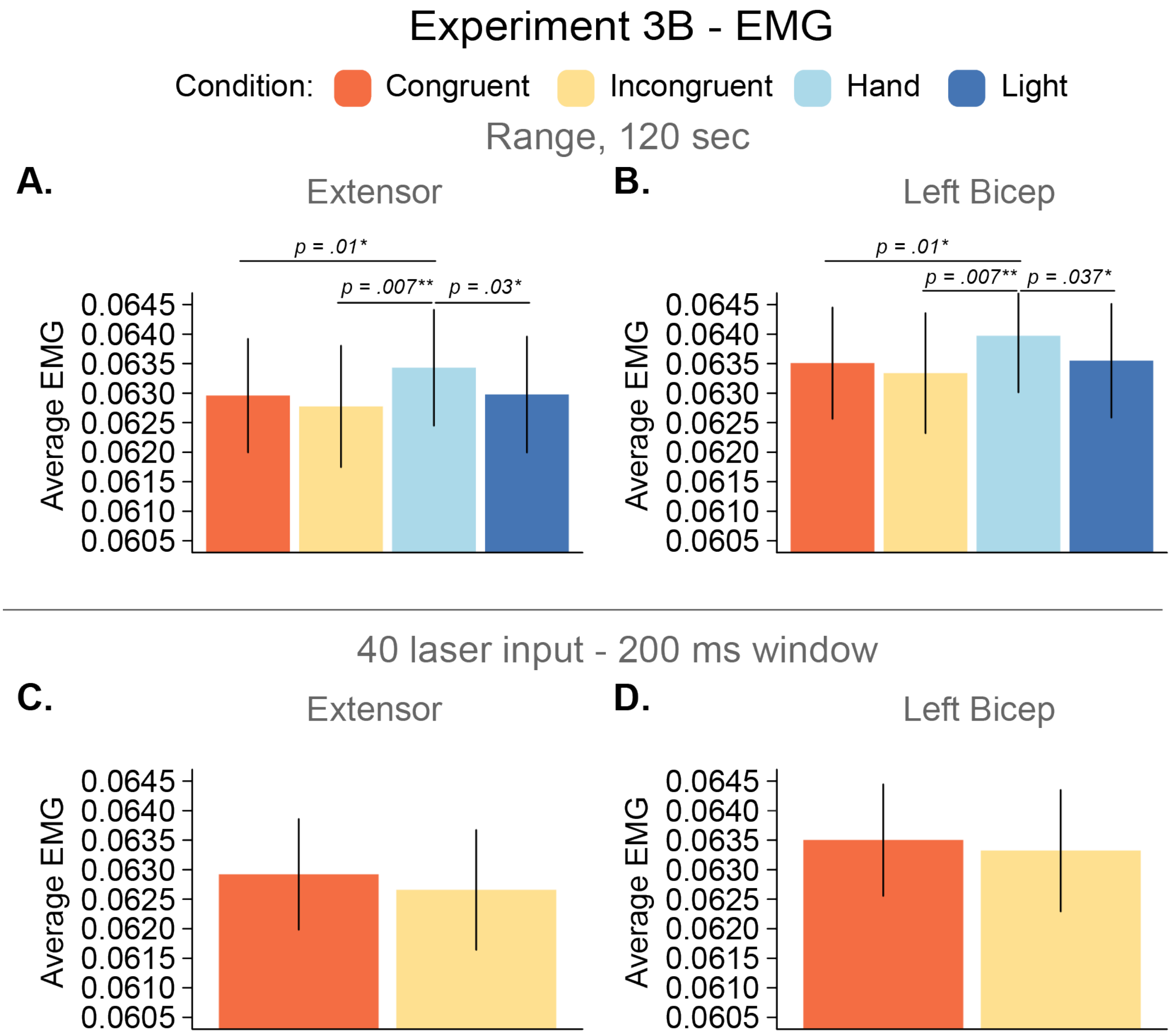

***Figure S8*. Bar plots EMG data, experiment 3B**. *The sample size is 29 since data from one subject were not recorded due to a device failure. (**A.**) The Left Extensor muscular averaged activity across the entire block (i.e., 120 sec) is shown for each condition. (**B**.) The Left Bicep muscular averaged activity across the entire block (i.e., 120 sec) is shown for each condition. (**C.**) The Left Extensor muscular averaged activity after each painful stimulation in the painful blocks, that is the average activity of 40 laser input, is shown. (**D.**) The Left Bicep muscular averaged activity after each painful stimulation in the painful blocks, that is the average activity of 40 laser input, is shown.*

Note: Error bars show standard error and 'rms' stands for 'root mean square'.

*Note: * = p < 0.05, ** = p < 0.01, *** = p < 0.001, uncorrected*

SECTION V – POST-HOC POOLED ANALYSIS

Pooled Analyses: EXPERIMENTS 1A+3A

(1) Participants

Data from the questionnaire-based Experiments 1A and 3A and from the proprioceptive drift Experiments 1B and 3B were combined to increase the robustness of the correlation findings. These experiments included the congruent and incongruent conditions from two different groups of participants, and therefore, the data could be pooled (30 M, 30 F, Age = 26.93 ± 4.70 SD, 50 right-handed, fluence = 64.182 mJ/mm^2 ± 19.39 SD).

EXPERIMENTS 1A+3A – Participants' descriptive				
What	***N***	***Handedness***	***Mean (± SD)***	***Median (min~max)***
VAS	60 (30M~30F)	50R~9L~1A	15.44 (±7.53)	15.65 (1.4~34.7)
Age	60 (30M~30F)	50R~9L~1A	26.93 (±4.7)	26 (18~45)
Fluence	60 (30M~30F)	50R~9L~1A	64.18 (±19.39)	64.99 (25.998~97.491)
Joule	60 (30M~30F)	50R~9L~1A	2.47 (±0.75)	2.5 (1~3.75)

***Table S23**. **Descriptive statistics participants, experiments 1A+3A.** Descriptive statistics of the participants data during experiment 3A are shown. SD = standard deviation, 1Q ~ 3Q =first quartile ~ third quartile. R = right-handed, L = left-handed, A = ambidextrous.*

(2) Questionnaire

EXPERIMENTS 1A+3A – Questionnaire descriptive				
Question	***Congruent***		***Incongruent***	
	Mean (± SD)	***Median (1Q~3Q)***	***Mean (± SD)***	***Median (1Q~3Q)***
S1	0.22 (±2.06)	1 (-2~2)	-0.95 (±1.99)	-1.5 (-3~1)
S2	0.12 (±2.08)	1 (-2~2)	-1 (±2.09)	-2 (-3~1)
S3	-1.43 (±1.85)	-2 (-3~0)	-1.45 (±1.87)	-2 (-3~0)
S4	-1.15 (±2.08)	-2 (-3~1)	-0.82 (±2)	-1 (-3~1)
S5	2.07 (±1.36)	2.5 (2~3)	-1.38 (±1.9)	-2 (-3~0)
S6	0.65 (±2.07)	1 (-1~2)	-1.28 (±1.9)	-2 (-3~0.25)
S7	-1.13 (±1.87)	-2 (-3~1)	-1.45 (±1.8)	-2 (-3~-0.75)
S8	-1.83 (±1.71)	-3 (-3~-1)	-2.02 (±1.7)	-3 (-3~-2)

***Table S24**. **Descriptive statistics questionnaire, experiments 1A+3A.** Descriptive statistics of the questionnaire values during experiments 1A+3A are shown. SD = standard deviation, 1Q ~ 3Q =first quartile ~ third quartile.*

EXPERIMENTS 1A+3A – Questionnaire analysis								
Comparison	***Statement***	***Statistic***	***95% CI***	***p value***	***p*** $\boldsymbol{value_{BH}}$	***Effect Size***	***Bayes Factor***	***Power (1-β)***
Congruent vs. Incongruent	*S1*	V = 759	1, 2	0***	0***	$r_C = 0.76$	$BF_{10} = 2757.81$	1
	S2	V = 681.5	1, 2.5	0***	0***	$r_C = 0.75$	$BF_{10} = 999.02$	1
	S5	V = 1317	3.5, 5	0***	0***	$r_C = 0.99$	$BF_{10} = 140328479674424$	1
	S6	V = 1019	2, 3.5	0***	0***	$r_C = 0.81$	$BF_{10} = 421452.88$	1

Table S25. Analysis questionnaire, experiments 1A+3A. *V = Wilcoxon sign rank statistic, BH = Bonferroni-Holm correction, r_C = paired rank-biserial correlation effect size, BF_{10} = bayes factor in favour of the alternative hypothesis, BF_{01} = bayes factor in favour of the null hypothesis.*
Importantly the Bonferroni-Holm correction was applied for 17 comparisons.
*Note: * = p < 0.05, ** = p < 0.01, *** = p < 0.001*

Ownership statements S1 and S2 were rated significantly higher in the congruent condition than in the incongruent condition (S1: V = 759, $p < 0.001$, $p_{BH} < 0.001$, 95% CI = [1, 2], $BF_{10} > 100$, $r_C = 0.76$; S2: V = 681.5, $p < 0.001$, $p_{BH} < 0.001$, 95% CI = [1, 2.5], $BF_{10} > 100$, $r_C = 0.75$). Similarly, the referral of pain statements S5 and S6 were significantly higher in the congruent condition (S5: V = 1317, $p < 0.001$, $p_{BH} < 0.001$, 95% CI = [3.5, 5], $BF_{10} > 100$, $r_C = 0.99$; S6: V = 1019, $p < 0.001$, $p_{BH} < 0.001$, 95% CI = [2, 3.5], $BF_{10} > 100$, $r_C = 0.81$).

Additionally, the percentage of people experiencing the N-RHI, which was assessed by scores ≥ 1 for S1, was 55%.

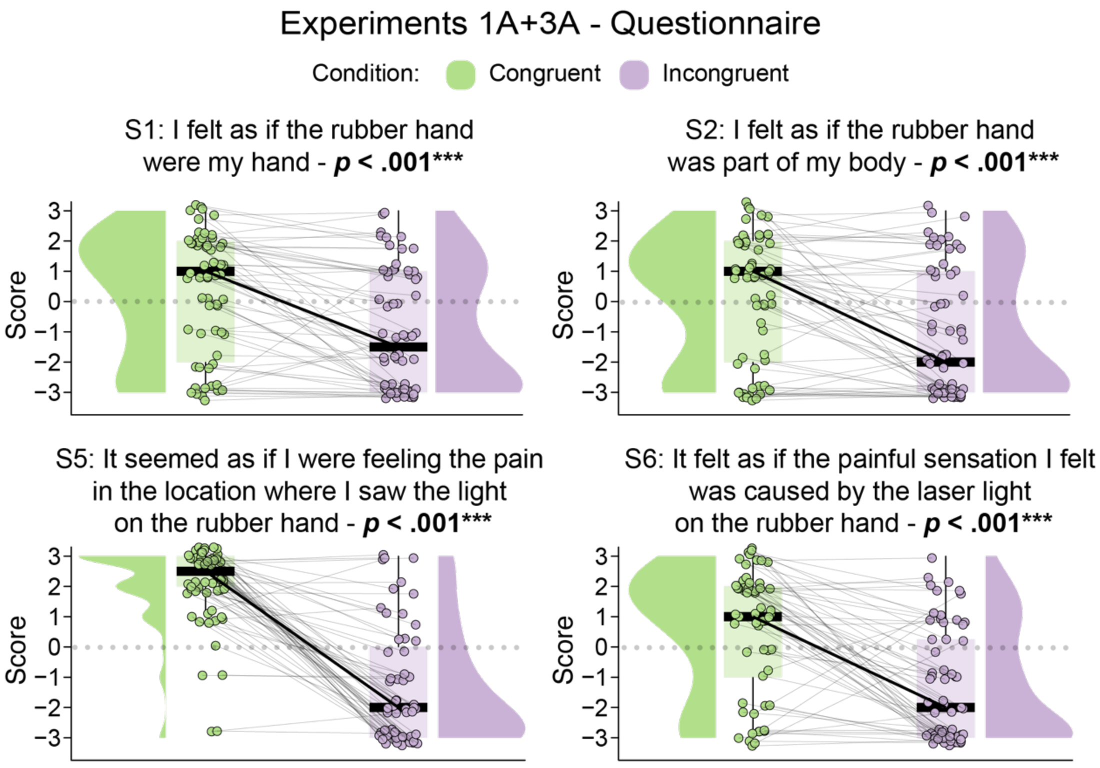

***Figure S9*. Raincloud plots questionnaire, experiments 1A+3A**. *Paired raincloud plots show individual data points and medians for each question in the questionnaire for Experiment 1A and 3A combined (N = 60). The questionnaire was administered on a Likert scale from -3 (not at all) to +3 (strongly agree).*
*Note: * = p < 0.05, ** = p < 0.01, *** = p < 0.001, uncorrected*

(3) VAS

EXPERIMENTS 1A+3A – VAS descriptive			
Variable	***Level***	***Mean (± SD)***	***Median (1Q~3Q)***
**Condition**	*Congruent*	23.62 (±13.78)	20.35 (14.58~28.8)
	Incongruent	24.07 (±15.51)	20.25 (15.25~30.62)
**Gender**	*Female*	24.15 (±14.76)	19.25 (16.25~25.6)
	Male	23.53 (±13.13)	21.88 (15.58~30.24)
**Block**	*Block 1*	24.34 (±14.73)	20.35 (16.17~30.62)
	Block 2	23.35 (±14.6)	20.3 (14.12~28.8)

***Table S26*. VAS Descriptive statistics, experiments 1A+3A.** *Descriptive statistics of the VAS values during experiment 1A+3A are shown. SD = standard deviation, 1Q ~ 3Q =first quartile ~ third quartile.*
*Note: * = p < 0.05, ** = p < 0.01, *** = p < 0.001*

EXPERIMENTS 1A+3A – VAS analysis							
Comparison	***Statistic***	***95% CI***	***p value***	***p value*** ***BH***	***Effect Size***	***Bayes Factor***	***Power (1-β)***
Congruent vs. Incongruent	t = -0.36	-2.95, 2.05	0.72	1	d_z = -0.05	BF_{01} = 6.65	0.06
Block 1 vs. 2	t = 0.79	-1.5, 3.47	0.43	1	d_z = 0.1	BF_{01} = 5.24	0.08
Female vs. Male	W = 471	-5.1, 6.4	0.762	1	r_G = 0.05	BF_{01} = 3.77	0.06

Table S27**. **Analysis VAS, experiments 1A+3A. *t = t test statistics, W = Wilcoxon rank sum statistic, BH = Bonferroni-Holm correction,* d_z *= Cohen* d_z *effect size,* r_G *= Glass biserial correlation effect size,* BF_{10} *= bayes factor in favour of the alternative hypothesis,* BF_{01} *= bayes factor in favour of the null hypothesis.*
Importantly the Bonferroni-Holm correction was applied for 3 comparisons.

There was no difference in pain ratings between conditions (t_{59} = -0.36, p = 0.72, p_{BH} = 1, 95% CI = [-2.95, 2.05], BF_{01} = 6.65, d_z = 0.05), nor between blocks (t_{59} = 0.79, p = .43, p_{BH} = 1, 95% CI = [-1.5, 3.47], BF_{01} = 5.24, d_z = 0.1), nor between genders (W = 471, p = 0.762, p_{BH} = 1, 95% CI = [-5.1,6.4], BF_{01} = 3.77, r_G = 0.05).

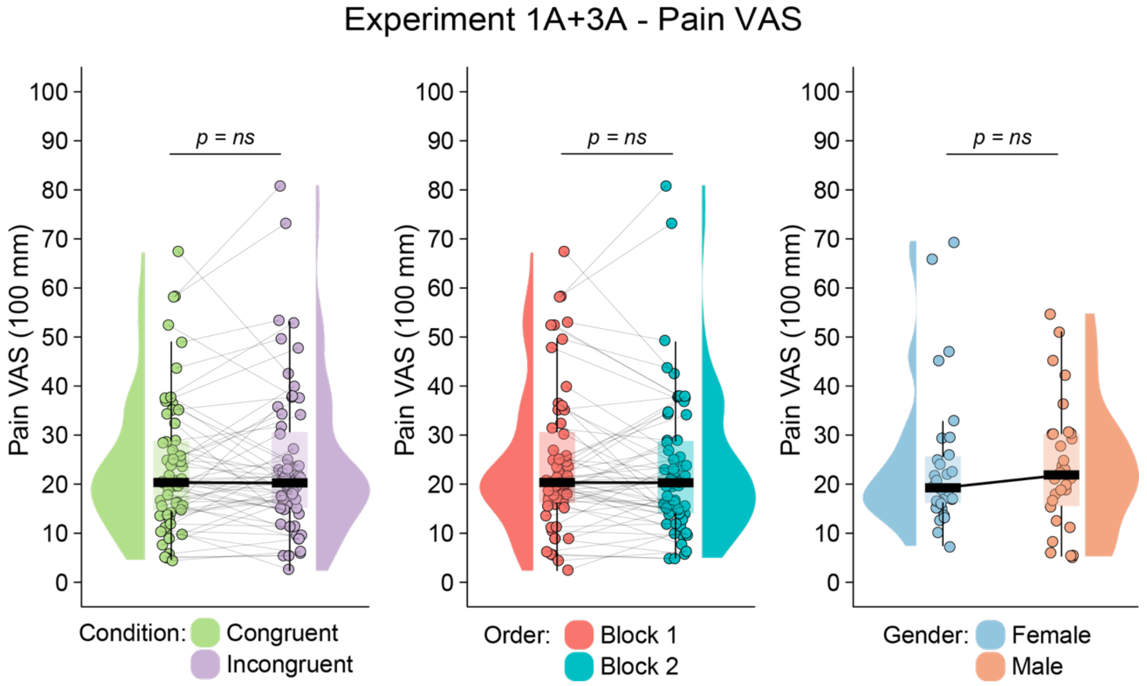

***Figure S10**. **Raincloud plots for VAS data, experiments 1A+3A**. Paired raincloud plots show individual data points in the Visual Analogue Scale (VAS) evaluation during experiment 1A+3A (N=60). (**A.**) The raincloud plot shows the VAS individual data points in the comparison between the two conditions, i.e., congruent vs. incongruent. (**B.**) The raincloud plot shows the VAS individual data points in the comparison between the two ordered blocks, i.e., the painful Block 1 vs. the painful Block 2. (**C.**) The raincloud plot shows the averaged VAS individual data points in the comparison between the two genders, i.e., Female vs. Male.*

(4) Correlations Analysis

Correlational analysis between ownership and referral of pain statements

Correlational analyses were run between ownership statements and referral of pain statements based on the differences in the scores when subtracting the incongruent condition from the congruent condition. There was a positive significant correlation between ownership statement S1 and referral of pain statement S5 ($r_s = 0.33$, $p = 0.01$, $p_{BH} = 0.177$, $BF_{10} = 2.21$), between the ownership statement S1 and the referral of pain statement S6 ($r_s = 0.67$, $p < 0.001$, $p_{BH} < 0.001$, $BF_{10} > 100$), and between the ownership statement S2 and the referral of pain statement S6 ($r_s = 0.44$, $p < 0.001$, $p_{BH} = 0.007$, $BF_{10} > 100$), but no significant correlation was found between the ownership statement S2 and the referral of pain statement S5 ($r_s = 0.19$, $p = 0.137$, $p_{BH} = 1$, $BF_{01} = 1.22$).

Correlational analysis between illusion ratings and pain VAS ratings

The correlational analyses were run separately for the two conditions. We did not find any significant statistical correlations between illusion-related questionnaire items and VAS pain ratings.

EXPERIMENTS 1A+3A – VAS and Questionnaire Correlational analysis

Comparison	***Correlation***	***p value***	***p value* BH**	***Bayes Factor***	***Power (1-β)***
S1 – S5 (Congruent-Incongruent)	$r_s = 0.33$	0.01*	0.177	$BF_{10} = 2.21$	0.441
S1 – S6 (Congruent-Incongruent)	$r_s = 0.67$	0***	0***	$BF_{10} = 24770.78$	0.989
S2 – S5 (Congruent-Incongruent)	$r_s = 0.19$	0.137	1	$BF_{01} = 1.22$	0.179
S2 – S6 (Congruent-Incongruent)	$r_s = 0.44$	0***	0.007**	$BF_{10} = 150.43$	0.71
S1 – VAS (Congruent-Incongruent)	$r_s = -0.07$	0.579	1	$BF_{01} = 1.49$	0.067
S2 – VAS (Congruent-Incongruent)	$r_s = -0.12$	0.367	1	$BF_{01} = 1.52$	0.096
S5 – VAS (Congruent-Incongruent)	$r_s = -0.14$	0.276	1	$BF_{01} = 2.51$	0.117
S6 – VAS (Congruent-Incongruent)	$r_s = -0.15$	0.251	1	$BF_{01} = 2.68$	0.125
S1 – VAS (Congruent)	$r_s = -0.05$	0.685	1	$BF_{01} = 3.04$	0.059
S2 – VAS (Congruent)	$r_s = -0.05$	0.715	1	$BF_{01} = 3.32$	0.057
S5 – VAS (Congruent)	$r_s = 0.05$	0.714	1	$BF_{01} = 3.11$	0.057
S6 – VAS (Congruent)	$r_s = 0.03$	0.81	1	$BF_{01} = 3.26$	0.053
S1 – VAS (Incongruent)	$r_s = -0.07$	0.618	1	$BF_{01} = 2.47$	0.063
S2 – VAS (Incongruent)	$r_s = -0.15$	0.243	1	$BF_{01} = 3.42$	0.128
S5 – VAS (Incongruent)	$r_s = 0$	0.99	1	$BF_{01} = 1.68$	0.049
S6 – VAS (Incongruent)	$r_s = -0.18$	0.167	1	$BF_{01} = 3.41$	0.161
S1 (Congruent-Incongruent) – VAS (Average)	$r_s = -0.09$	0.516	1	$BF_{01} = 2.03$	0.073
S2 (Congruent-Incongruent) – VAS (Average)	$r_s = 0$	0.985	1	$BF_{01} = 3.36$	0.05
S5 (Congruent-Incongruent) – VAS (Average)	$r_s = 0$	0.975	1	$BF_{01} = 2.2$	0.05
S6 (Congruent-Incongruent) – VAS (Average)	$r_s = 0.11$	0.422	1	$BF_{01} = 3.41$	0.086

Table S28*. *Correlational analyses between questionnaire and VAS, experiments 1A+3A. *r_s = Spearman rho, BH = Bonferroni-Holm correction, BF_{10} = bayes factor in favour of the alternative hypothesis, BF_{01} = bayes factor in favour of the null hypothesis.*
Importantly the Bonferroni-Holm correction was applied for 20 comparisons.
*Note: * = p < 0.05, ** = p < 0.01, *** = p < 0.001*

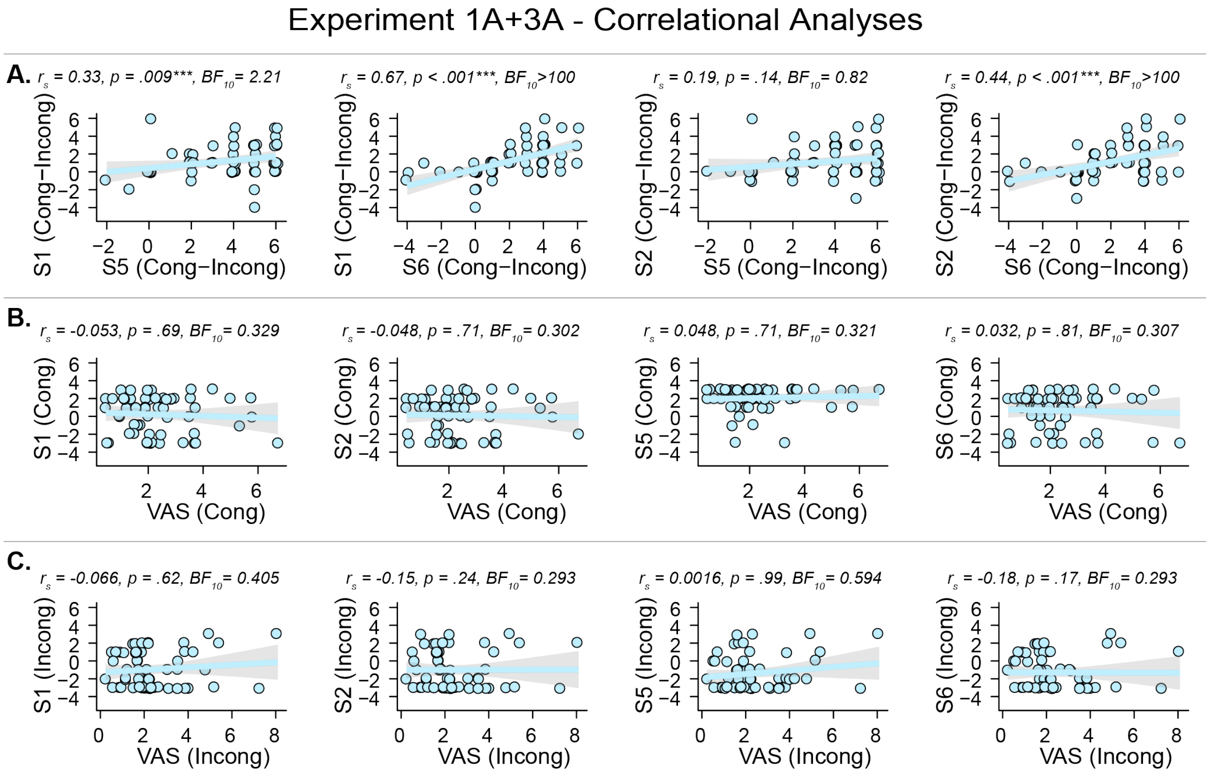

***Figure S11*. *Correlational plots, experiments 1A+3A*.** *Linear regressions are shown for visualization purposes and the Spearman correlation (r_S) was run in the pooled data of experiments 1A and 3A (N = 60). (**A.**) Correlational analyses between ownership questions (S1 and S2) and referral of pain questions (S5 and S6) for the difference between congruent and incongruent conditions (Cong-Incong). (**B.**) Correlational analyses between visual analogue scale (VAS) for pain perception and the ownership questions and the referral of pain questions in the congruent condition (Cong). (**C.**) Correlational analyses between visual analogue scale (VAS) for pain perception and the ownership questions and the referral of pain questions in the incongruent condition (Incong).*

*Note: * = p < 0.05, ** = p < 0.01, *** = p < 0.001, uncorrected*

Pooled Analyses: EXPERIMENTS 1B+3B

(1) Participants

EXPERIMENTS 1B+3B – Participants descriptive				
What	***N***	***Handedness***	***Mean (± SD)***	***Median (min~max)***
VAS	60 (25M~35F)	59R~0L~1A	13.17 (±5.98)	13.45 (2.4~35.2)
Age	60 (25M~35F)	59R~0L~1A	25.87 (±4.32)	25 (19~36)
Fluence	60 (25M~35F)	59R~0L~1A	60.77 (±19.36)	61.74 (25.998~103.99)
Joule	60 (25M~35F)	59R~0L~1A	2.34 (±0.74)	2.38 (1~4)

Table S29*. *Descriptive statistics participants, experiments 1B+3B. *Descriptive statistics of the participants data during experiments 1B+3B are shown. SD = standard deviation, 1Q ~ 3Q =first quartile ~ third quartile. R = right-handed, L = left-handed, A = ambidextrous.*

(2) Proprioceptive Drift

EXPERIMENTS 1B+3B – Drift descriptive			
Variable	***Level***	***Mean (± SD)***	***Median (1Q~3Q)***
**Condition**	*Congruent*	1.07 (±2.55)	0.58 (-0.38~2.12)
	Incongruent	0.01 (±2)	0.25 (-1.5~1.17)

Table S30*. *Descriptive statistics proprioceptive drift, experiments 1B+3B. *Descriptive statistics of the proprioceptive drift data during experiments 1B+3B are shown. SD = standard deviation, 1Q ~ 3Q =first quartile ~ third quartile.*

EXPERIMENTS 1B+3B – Drift analysis						
Comparison	***Statistic***	***95% CI***	***p value***	***Effect Size***	***Bayes Factor***	***Power (1-β)***
Congruent vs. Incongruent	$t = 4.12$	0.55, 1.58	0***	$d_z = 0.53$	$BF_{10} = 184$	0.8

Table S31*. *Analysis proprioceptive drift, experiments 1B+3B. *t = t test statistics, d_z = Cohen d_z effect size, BF_{10} = bayes factor in favour of the alternative hypothesis, BF_{01} = bayes factor in favour of the null hypothesis.*
*Note: * = p < 0.05, ** = p < 0.01, *** = p < 0.001*

The proprioceptive drift toward the rubber hand was significantly higher in the congruent condition than in the incongruent condition ($t_{59} = 4.2$, $p < 0.001.72$, 95% CI = [0.55, 1.58], $BF_{10} > 100$, $d_z = 0.532$) (Figure S18).

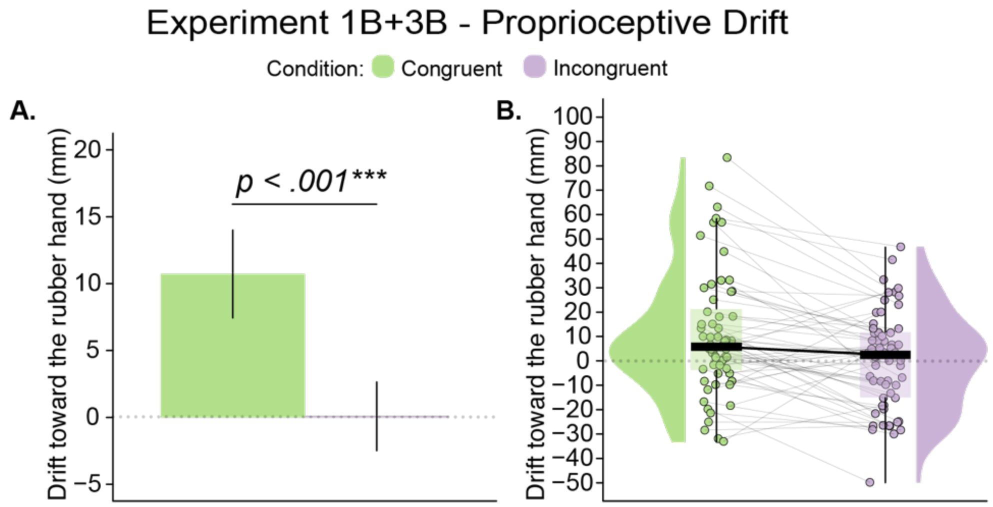

***Figure S12**. **Plots proprioceptive drift, experiments 1B+3B**. Here we combined data from experiments 1B and 3B, therefore we analyzed data from 60 subjects. (A.) Barplots and standard errors are shown. (B.) Paired raincloud plots show individual data points and medians of the proprioceptive drift task.*

*Note: * = p < 0.05, ** = p < 0.01, *** = p < 0.001*

(3) VAS

EXPERIMENTS 1B+3B – VAS descriptive			
Variable	***Level***	***Mean (± SD)***	***Median (1Q~3Q)***
**Condition**	*Congruent*	21.93 (±12.3)	18.2 (13.55~27.25)
	Incongruent	21.07 (±11.1)	18.38 (12.5~26.98)
**Gender**	*Female*	20 (±11.16)	18.07 (11.43~24.81)
	Male	23.6 (±11.53)	23.35 (15.3~29.5)
**Block**	*Block 1*	22.28 (±13.46)	19.9 (13.6~27.4)
	Block 2	21.1 (±13.29)	17.25 (11.88~27.25)
	Block 3	22.69 (±13.44)	17.7 (13.25~31.75)
	Block 4	21.74 (±13.15)	19.55 (13.32~29.7)
	Block 5	20.63 (±12.23)	17.15 (11.85~25.6)
	Block 6	20.54 (±12.6)	16.5 (10.83~23.52)

***Table S32**. **VAS Descriptive statistics, experiments 1B+3B.** Descriptive statistics of the VAS values during experiment 1B+3B are shown. SD = standard deviation, 1Q ~ 3Q =first quartile ~ third quartile.*

EXPERIMENTS 1B+3B – VAS analysis							
Comparison	***Statistic***	***95% CI***	***p value***	***p value* BH**	***Effect Size***	***Bayes Factor***	***Power (1-β)***
Congruent vs. Incongruent	V = 1182.5	0, 2.57	0.049*	0.783	r_C = 0.29	BF_{10} = 0.27	0.35
Block 1 vs. 2	V = 1051.5	-0.6, 3.05	0.209	1	r_C = 0.19	BF_{01} = 4.7	0.17
Block 1 vs. 3	*t* = -0.32	-2.97, 2.16	0.752	1	d_z = -0.04	BF_{01} = 6.75	0.06
Block 1 vs. 4	V = 1025	-1.5, 3.55	0.418	1	r_C = 0.12	BF_{01} = 6.7	0.1
Block 1 vs. 5	V = 1076	-0.95, 3.35	0.236	1	r_C = 0.18	BF_{01} = 3.37	0.15
Block 1 vs. 6	V = 1165	-0.1, 4.3	0.066	0.986	r_C = 0.27	BF_{01} = 2.97	0.31
Block 2 vs. 3	V = 575	-3.55, -0.15	0.03*	0.508	r_C = -0.33	BF_{10} = 0.39	0.43
Block 2 vs. 4	V = 681.5	-3, 0.4	0.086	1	r_C = -0.26	BF_{01} = 6.45	0.28
Block 2 vs. 5	V = 947	-2.35, 2.5	0.814	1	r_C = 0.03	BF_{01} = 6.68	0.05
Block 2 vs. 6	V = 969.5	-1.7, 2.65	0.688	1	r_C = 0.06	BF_{01} = 6.46	0.06
Block 3 vs. 4	*t* = 0.74	-1.63, 3.53	0.463	1	d_z = 0.1	BF_{01} = 5.46	0.08
Block 3 vs. 5	*t* = 1.69	-0.39, 4.51	0.097	1	d_z = 0.22	BF_{01} = 1.87	0.21
Block 3 vs. 6	*t* = 1.72	-0.36, 4.66	0.092	1	d_z = 0.22	BF_{01} = 1.79	0.22
Block 4 vs. 5	V = 995	-1.15, 2.6	0.406	1	r_C = 0.12	BF_{01} = 4.76	0.1
Block 4 vs. 6	V = 980	-1.2, 2.5	0.473	1	r_C = 0.11	BF_{01} = 3.94	0.09
Block 5 vs. 6	V = 801	-2, 1.5	0.673	1	r_C = -0.06	BF_{01} = 7.06	0.06
Female vs. Male	W = 523	-1.75, 8.42	0.204	1	r_G = -0.2	BF_{01} = 2.04	0.180

***Table S33**. **Analysis VAS, experiments 1B+3B.** t = t test statistics, V = Wilcoxon sign rank statistic, W = Wilcoxon rank sum statistic, BH = Bonferroni-Holm correction, d_z = Cohen d_z effect size, r_C = paired rank-biserial correlation effect size, r_G = Glass biserial correlation effect size, BF_{10} = bayes factor in favour of the alternative hypothesis, BF_{01} = bayes factor in favour of the null hypothesis.*
Importantly the Bonferroni-Holm correction was applied for 17 comparisons.
*Note: * = p < 0.05, ** = p < 0.01, *** = p < 0.001*

The intensity of pain was rated higher in the congruent condition as compared to the incongruent condition, but it did not survive multiple comparisons (V = 1182.5, p = 0.049, p_{BH} = 0.783, 95% CI = [0, 2.57], BF_{10} = 0.27, r_C = 0.29). The pain rating was significantly higher in Block 2 as compared to Block 3, but it did not survive multiple comparisons (V = 575, p = 0.03, p_{BH} = 0.508, 95% CI = [-3.55, -0.15], BF_{10} = 0.39, r_C = -0.33). There was no difference in the perceived pain during the experiment between females and males (V = 523, p = 0.204, p_{BH} = 1, 95% CI = [-1.75, 8.42], BF_{01} = 2.04, r_G = -0.2).

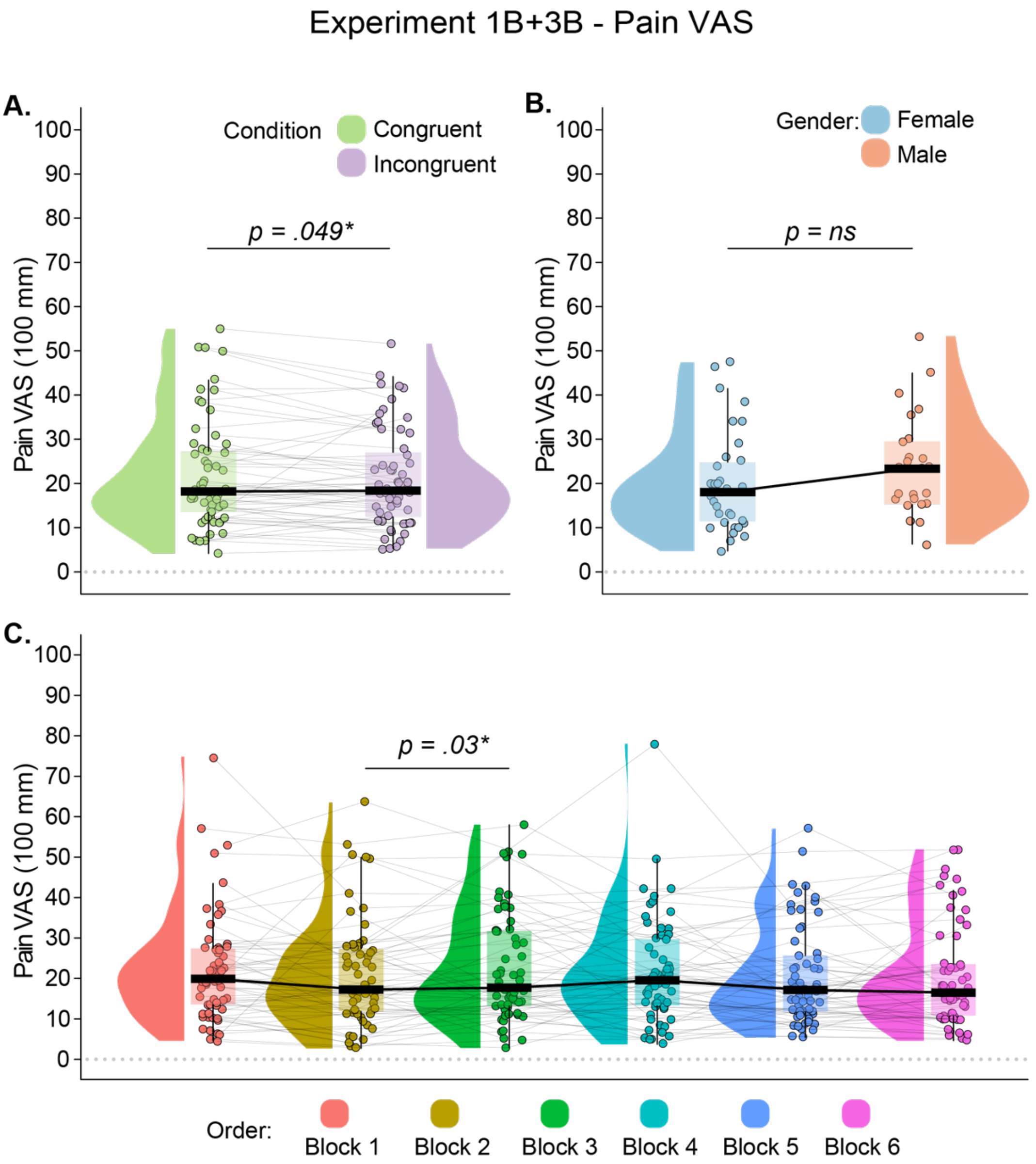

***Figure S13*. *Raincloud plots for VAS data, experiments 1B+3B*.** *Paired raincloud plots show individual data points in the Visual Analogue Scale (VAS) evaluation of experiment 1B and 3B pooled data (N= 60). (**A.**) The raincloud plot shows the VAS individual data points in the comparison between the two conditions, i.e., congruent vs. incongruent. (**B.**) The raincloud plot shows the averaged VAS individual data points in the comparison between the two genders, i.e., Female vs. Male. (**C.**) The raincloud plot shows the VAS individual data points in all painful blocks of the experiments, i.e., Block 1 vs. Block 2 vs. Block 3 vs. Block 4 vs. Block 5 vs. Block 6.*

*Note: * = p < 0.05, ** = p < 0.01, *** = p < 0.001*

(4) Correlation analysis

Correlational analysis between proprioceptive drift and pain VAS ratings.

No significant correlation was found between proprioceptive drift and the rated pain intensity in the congruent condition ($r_s = 0.06$, $p = 0.647$, $p_{BH} = 1$); however, a significant positive correlation between proprioceptive drift and VAS pain ratings was found in the incongruent condition ($r_s = 0.26$, $p = 0.045$, $p_{BH} = 0.179$, $BF_{10} = 3.08$), but it did not survive multiple comparisons.

EXPERIMENTS 1B+3B – VAS and Drift Correlational analysis					
Comparison	***Correlation***	***p value***	***p value BH***	***Bayes Factor***	***Power (1-β)***
Drift –VAS (Congruent-Incongruent)	$r_s = 0.04$	0.79	1	$BF_{01} = 3.19$	0.053
Drift – VAS (Congruent)	$r_s = 0.06$	0.647	1	$BF_{01} = 0.81$	0.061
Drift – VAS (Incongruent)	$r_s = 0.26$	0.045*	0.179	$BF_{10} = 3.08$	0.289
Drift (Congruent-Incongruent) – VAS (Average)	$r_s = 0.01$	0.962	1	$BF_{01} = 3.32$	0.05

Table S34*. *Correlational analyses between proprioceptive drift and VAS, experiments 1B+3B. *r_s = Spearman rho, BH = Bonferroni-Holm correction, BF_{10} = bayes factor in favour of the alternative hypothesis, BF_{01} = bayes factor in favour of the null hypothesis.*
Importantly the Bonferroni-Holm correction was applied for 4 comparisons.
*Note: * = p < 0.05, ** = p < 0.01, *** = p < 0.001*

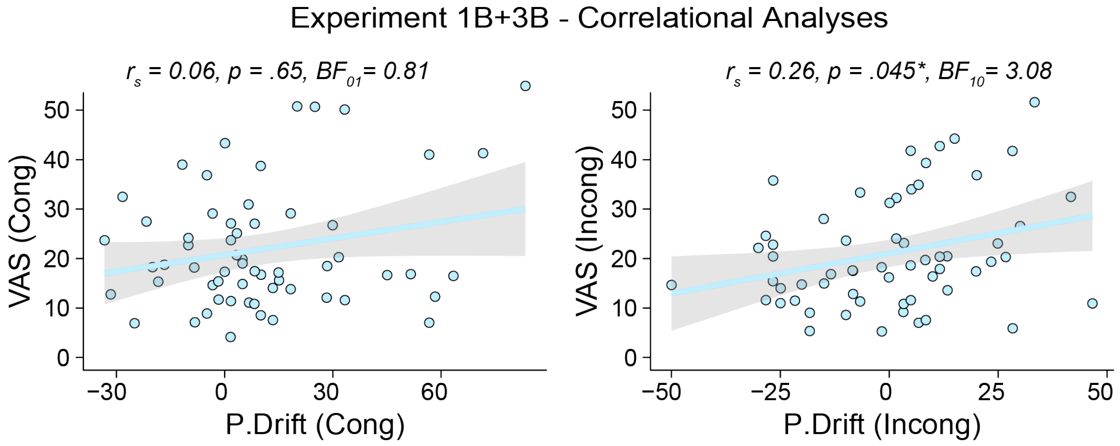

Figure S14. Correlational plots, experiments 1B+3B. *Linear regression plots are shown for visualization purposes and the Spearman correlation (r_s) was run. The analyses were run on the pooled data of experiments 1B and 3B (N=60). The left panel shows the correlational analysis between proprioceptive drift and VAS in the congruent condition (Cong), whereas in the right panel the correlational analysis between proprioceptive drift and VAS in the incongruent condition (Incong) is shown.*
*Note: * = p < 0.05, ** = p < 0.01, *** = p < 0.001, uncorrected*

Conclusions for post hoc pooled analysis.

The results of the analysis of the pooled dataset were consistent with the individual results obtained from Experiments 1A, 1B, 3A and 3B. We also observed a correlation between the ownership and referral of pain statements, similar to the correlation observed between the ownership and referral of touch statements in the classic tactile RHI [3,4]. Furthermore, the observation that pain VAS and the N-RHI measures are uncorrelated was further strengthened.

Bibliography

[1] King BM, Rosopa PJ, Minium EW. Statistical reasoning in the behavioral sciences. John Wiley & Sons, 2018.

[2] Lakens D. Calculating and reporting effect sizes to facilitate cumulative science: a practical primer for t-tests and ANOVAs. Front Psychol 2013;4. doi:10.3389/fpsyg.2013.00863.

[3] Longo MR, Schüür F, Kammers MPM, Tsakiris M, Haggard P. What is embodiment? A psychometric approach. Cognition 2008;107:978–998. doi:10.1016/j.cognition.2007.12.004.

[4] Reader AT, Trifonova VS, Ehrsson HH. The Relationship Between Referral of Touch and the Feeling of Ownership in the Rubber Hand Illusion. Front Psychol 2021;12:629590. doi:10.3389/fpsyg.2021.629590.
